# The logic of the floral transition: Reverse-engineering the switch controlling the identity of lateral organs

**DOI:** 10.1371/journal.pcbi.1005744

**Published:** 2017-09-20

**Authors:** Jean-Louis Dinh, Etienne Farcot, Charlie Hodgman

**Affiliations:** 1 Centre for Plant Integrative Biology, School of Biosciences, University of Nottingham, Sutton Bonington, United Kingdom; 2 School of Mathematical Sciences, University of Nottingham, Nottingham, United Kingdom; Universidad Nacional Autónoma de México, MEXICO

## Abstract

Much laboratory work has been carried out to determine the gene regulatory network (GRN) that results in plant cells becoming flowers instead of leaves. However, this also involves the spatial distribution of different cell types, and poses the question of whether alternative networks could produce the same set of observed results. This issue has been addressed here through a survey of the published intercellular distribution of expressed regulatory genes and techniques both developed and applied to Boolean network models. This has uncovered a large number of models which are compatible with the currently available data. An exhaustive exploration had some success but proved to be unfeasible due to the massive number of alternative models, so genetic programming algorithms have also been employed. This approach allows exploration on the basis of both data-fitting criteria and parsimony of the regulatory processes, ruling out biologically unrealistic mechanisms. One of the conclusions is that, despite the multiplicity of acceptable models, an overall structure dominates, with differences mostly in alternative fine-grained regulatory interactions. The overall structure confirms the known interactions, including some that were not present in the training set, showing that current data are sufficient to determine the overall structure of the GRN. The model stresses the importance of relative spatial location, through explicit references to this aspect. This approach also provides a quantitative indication of how likely some regulatory interactions might be, and can be applied to the study of other developmental transitions.

## Author summary

Complex organisms develop following a genetic program specifying how their cells differentiate into organs with different specialized functions. Details of these mechanisms are incomplete, and aspects might not be correct. These genetic programs can be represented by simple computer programs processing input data into outputs, as a way of finding out more about cell differentiation. Our strategy has entailed generating and analyzing many programs whose outcomes do not conflict with our current biological knowledge for any of the documented combinations of inputs. Initial brute-force construction and evaluation of smaller networks was not entirely successful. We go on to present arithmetic arguments which show that it is computationally unfeasible for larger networks to check all possible programs for complying with biological knowledge. Hence, we have employed an approach inspired by biological evolution, namely genetic programming, with refinements to favor the most likely and not overly complex programs. Our test case is the program used by plants to produce flowers (a prerequisite for fruits and seeds), which is commercially very important. Our technique showed that the spatial distribution of cells was crucial and most of the presumed mechanism occurred in all programs, and was able both to point out probable gaps in our biological knowledge and to suggest suitable additions. Furthermore, this approach can be applied to other biological developmental programs.

## Introduction

Computational approaches have become routinely used in the study of gene regulatory networks [1,2]. One of the fundamental key outcomes of gene-network activity is specification of the differentiated cell types during development that lead to different tissues and organs. To address this particular question, computational models have to capture the unfolding, both in time and space, of the program embodied by interactions between genes, transcription factors and other molecular complexes. This necessity to describe spatio-temporal patterns of gene activity entails an important computational cost. In addition, laboratory techniques do not yet exist that determine the precise spatio-temporal patterns of gene expression of multiple genes in a single data set, making the development of such models very difficult. This paper proposes tools designed to represent the specification of new cell identities during development, and to fit models against incomplete data. This work focuses on the network controlling the Shoot Apical Meristem (SAM) during the floral transition, see below. However, the methods aim to be applicable to other systems involving cell differentiation and the underlying spatial patterning of biological tissues.

Flowers are the reproductive organs of plants. Therefore, their formation is crucial for reproductive success. From a developmental perspective, flower formation starts with the triggering of specific pathways in the founder cells of lateral organs (i.e. leaves initially), so that they develop into flowers instead. This developmental switch is called the floral transition. It is one of many aspects of cell-fate specification in the Shoot Apical Meristem, which comprises multiple tissues, each with their own gene-expression profile but all produced from a single stem-cell population. This early specification of cell types, through the interactions between genes and hormones, enables newly formed tissues to later develop into all the aerial parts of a plant [3,4]. The transition goes through three well-characterized stages, starting with a vegetative meristem, which produces leaves. Upon the trigger by the appearance of the protein FT, this meristem becomes an inflorescence meristem, from which floral meristems appear that produce flowers.

While the pathways involved in the floral transition have been reviewed [5,6] and modelled (using Ordinary Differential Equations (ODEs) [7–9] and neural networks [10]), these studies give little if any attention to the spatial organization of the SAM and do not include any representation of space. The side effects of this simplification obviously include the inability to explain how the spatial organization of the SAM is acquired, but also the prediction of unrepresentative gene-expression profiles, because the gene expression measurements have come from multiple cell types. This potentially leads to the consideration of combinations of regulatory interactions that cannot actually occur *in vivo*, because the genes involved are not, in reality, expressed in the same cells.

The present study focuses on how the gene-regulatory network of the SAM is able to determine the transition of its daughter cells into stem, leaf, flower or other cell types, based on environmental and positional cues. To address the lack of spatial information found in previously published studies, a novel approach was required. We therefore propose a modelling framework which includes an explicit representation of space in terms of the organs and tissues found in different parts of the SAM. This is analogous to studies of invertebrate development [11,12].

Regulations known from the literature may be ambiguous, so the proposed methodology comprises a method for the inference of models, based on experimental data. This entailed generating a compendium of published *in situ* hybridization (ISH) experiments, to describe groups of jointly expressed genes. Models deemed plausible had to reproduce both the observed patterns of co-expression and the known developmental transitions. This offers the potential to explore alternatives to current thinking about the regulatory mechanisms and predict novel regulatory interactions for laboratory testing.

If ODE modelling is used, the number of possible alternative regulatory interactions, even among a small number of genes, would lead to unfeasibly long parameter-estimation times. This is because the uncertainty on interactions leads to a very large number of candidate model topologies, for each of which, given the expected sloppiness of most models [13], parameters have to be estimated multiple times. Based on typical estimation times and current computing capability, this would take several years of computation for the complexity of the model considered here. However, a formalism particularly well suited to this task is Boolean modelling, which naturally handles binary (on or off) variables that accord with the resolution of the ISH data. For a brief introduction to Boolean models, please refer to S1 Text. Even though Boolean models are lightweight, the space of possible models for a given set of genes remains computationally expensive to explore. In simple cases, this “model” space can be explored through exhaustive searches, but it quickly becomes intractable as the number of possible regulatory interactions increases. In more complex cases, heuristic techniques are required. In this work, a genetic programming algorithm has been employed to find suitable models that explain all observed data.

Boolean network models have been used successfully to study developmental processes, such as floral development [14], which directly follows the floral transition. By representing genes as binary variables influencing each other, they enable us to run simulations and find steady states of the system. These steady states can then be interpreted as cell identities or expression profiles. The idea of matching biological observations to steady states in not new: the logical rules built by Espinosa-Soto and colleagues resulted in steady-states matching biological observations. This work describes a related process: building up the logical rules from the biological observations. It is similar to what has been done by La Rota *et al*. for the regulatory network controlling sepal formation [15].

Genetic algorithms have previously been used in conjunction with Boolean modelling [11,12]. These methods operate on Boolean models at the level of truth tables, whereas genetic programming operates at the level of equations. While truth tables can always be generated from equations and equations can be factorized from truth tables, working on equations has several benefits: factorizing equations is more expensive than deriving truth tables, equations are human-readable, and constraints of complexity can be enforced on them.

This work has shown, for the network controlling SAM identity, that an exhaustive search of all possible regulatory interactions is prohibitive. Restricting the search to models supported by the published regulatory networks explains the steady states but, when attempting to explain the dynamic transitions between them, result in many ambiguous regulatory events. Using genetic programming to find models that correspond to the ISH data and known cell type transitions reduced the ambiguity almost entirely, and identified other regulatory interactions that have been independently confirmed in other published work.

## Results

**Fig 1:**
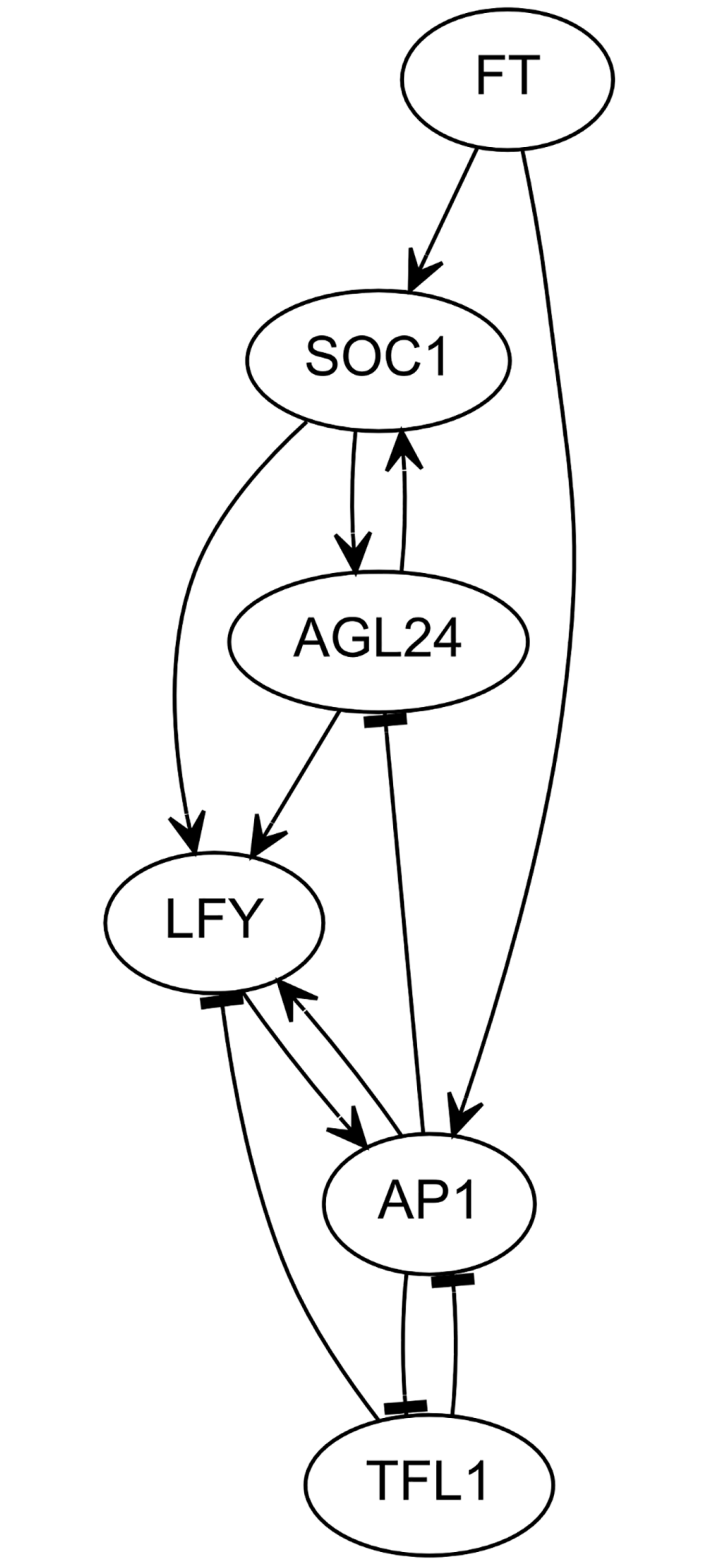
Common representation of the core regulatory network controlling SAM identity. Nodes represent genes and edges represent regulatory interactions. V-shaped and T-shaped arrow heads respectively denote activation and repression by the regulatory nodes.

The most common representation of the core regulatory-network [5] is shown in Fig 1, though other regulatory components have also been reviewed by Liu et al.[6]. As a necessary first verification, one needs to assess whether this topology is sufficient to generate the observed patterns of gene expression, or if new regulators or interactions are required. As detailed below, a given topology, or regulatory graph, can be achieved by a large number of distinct models and one needs to determine whether at least one of them is able to generate the required expression patterns. In some cases, all the potential models can be listed exhaustively, but it will soon become clear that, in the general case, the space to explore is too large to allow for an exhaustive search.

### The cost of running an exhaustive search on the whole space of possible models is prohibitive

Classically, three meristematic identities are distinguished: vegetative, inflorescence and floral [3,4], and are normally defined by five main genes. *SOC1* and *AGL24* are markers of the inflorescence identity, and *LFY* and *AP1* of the floral identity [16–19], while *TFL1* inhibits the floral identity [16,20] and is a marker of vegetative identity (the inflorescence also expresses *TFL1* though, which can be attributed to the inflorescence conserving some vegetative traits). A sixth gene, *FT*, encodes a mobile protein that is synthesized in leaves, moves to the SAM through the phloem [21] and triggers the transition from the vegetative to the inflorescence and floral identities. However, owing to a memory effect, FT is not needed to maintain the inflorescence and floral identities after the floral transition [4]. Each meristematic identity has a characteristic expression profile (Table 1). The question arises of whether or not there are any other regulatory combinations of these genes than those reviewed in the literature that result in the same set of identities.

**Table 1:** Genes expressed in the three classical meristematic identities.

Vegetative	*TFL1* [4]
Inflorescence	*(*FT*)*, *SOC1* [4], *AGL24* [4], *TFL1* [4,22]
Floral	*(*FT*)*, *AP1* [4,22], *LFY* [22]

The number of models to examine is a function of the numbers of input nodes (nodes with no inbound regulation) and internal nodes (nodes with inbound regulations). As discussed in more detail in S1 Text, a Boolean model is a map acting on the set of all possible states (combinations of “on”/”off” status of each node) of the system and can be thought of as a logic program. From an initial state, this map determines the content of its “successor” state, with repeated references to it resulting in dynamical evolution of the system. If a state is identical to its successor, then it is a steady state. There are 6 nodes in total, so there are *2*^*6*^ = *64* possible Boolean states of the system. To define a model of this system, a successor must be defined for each of these 64 states. The behavior of input nodes is fixed, so successors are uniquely characterized by the behaviors of the five internal nodes. Looking naively at the full set of all Boolean models, there are therefore *2*^*5*^ = *32* possible choices of successor for each of the 64 states, i.e. *32*^*64*^ = *2*^*320*^ ≃ *2*.*10*^*96*^ potential models. This is more than the estimated number of atoms in the observable universe, which is ~10^80^. Even with a computer able to check 10 billion models per second, it would still take ~6.10^78^ years. This quick estimate shows that a brute force approach is impractical and that one needs to constrain the search space using prior biological knowledge.

### The topology summarized by Fornara et al. can explain the steady states but not the dynamic behavior

The first, obvious, constraint on the search space is to exclude models containing regulatory interactions that are not backed by any biological evidence. As an added benefit, should solutions be found, this would demonstrate that the set of evidence-backed interactions is comprehensive enough to explain the behavior of the system. In an attempt to find a reasonably sized set of regulatory interactions that can explain the behavior of the system, the Fornara *et al*. network [5] has been used as the main source of prior knowledge, without any additions from Liu *et al*. [6]. This set can be determined very cheaply, as it is comprised of all the models whose truth tables follow a pattern depending solely on the required steady states and the topology of the network (see S2 Text).

In this work, the exhaustive search produced a set of 262,144 models exhibiting the required steady states. This topology is therefore sufficient to explain the steady states of the system. However, it cannot reproduce state transitions undergone by the real biological system during development and, astonishingly, does not include the activation of *SOC1* by FT (see S1 Fig), the known trigger for flowering.

**Fig 2:**
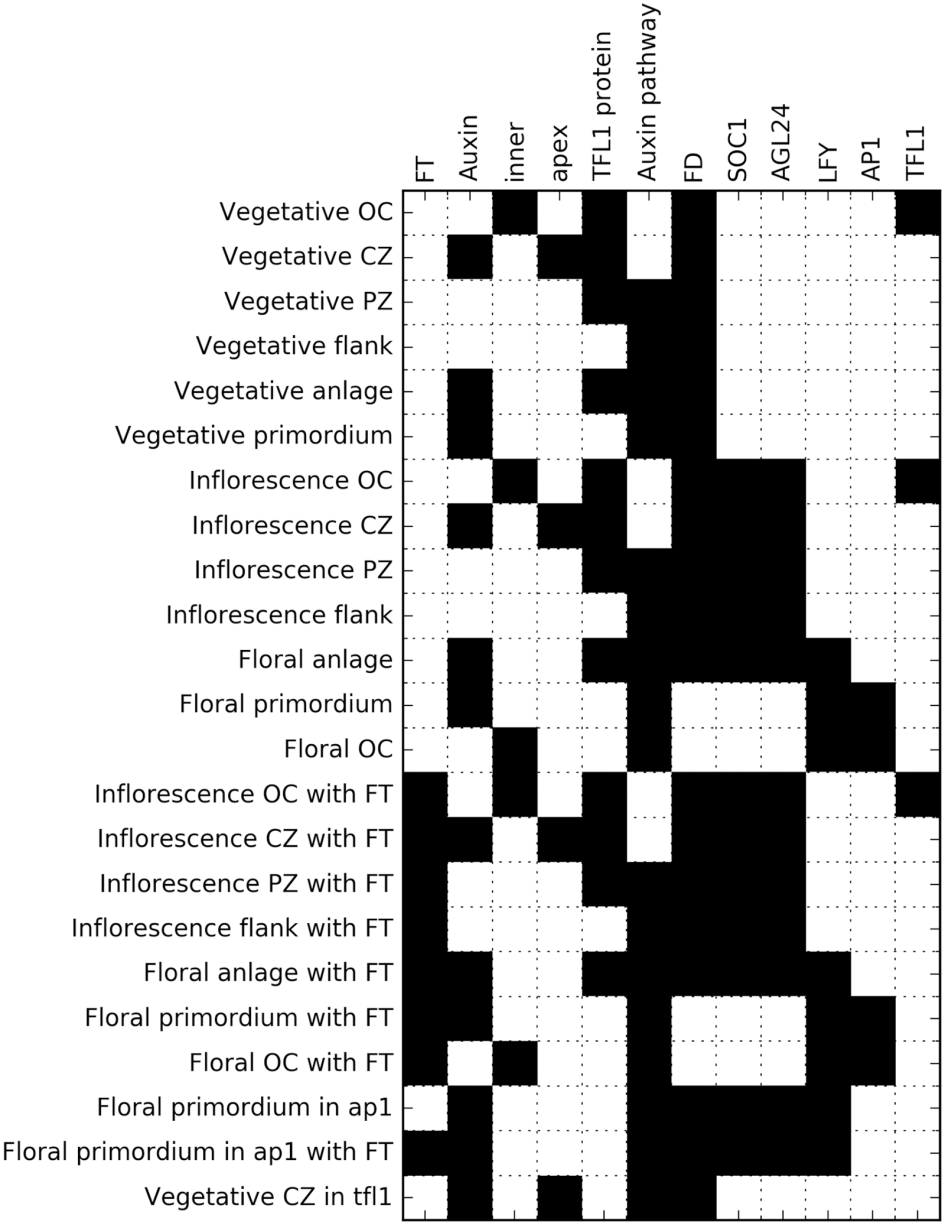
Matrix of gene expressions in cell populations identified from ISH pictures. Rows correspond to cell populations and columns to chemical species or other variables. A black square means a species is present or a variable is on in the associated tissue.

In our modelling framework, we describe transitions as the given of an initial steady state I, a perturbation P to be applied to that steady state, and a final steady state F, resulting from the spontaneous evolution of the system following the perturbation. Both I and F correspond to one of the cell identities described in Fig 2 and P to the toggling of one or a few variables representing non cell-autonomous species. These species are therefore effectively the triggers of the transitions from the modelling perspective. The associated biological interpretation is that non cell-autonomous species form spatial patterns in the SAM that are constantly perturbed by growth and cell divisions. This causes cells to enter some patterns and exit others, as those patterns reorganize. The topology by Fornara *et al*. lacks a trigger with a pattern matching the position of floral primordia, and hence cannot explain the dynamic behavior of the SAM.

### Addition of two interactions yields models that are able to mimic the changes in cell identities

The failure of this exhaustive search to explain dynamical behavior requires the model to be enlarged. Two interactions from Liu et al., namely AP1 → SOC1 and Auxin → LFY, were added on the basis of parsimony, the intuitive fact that they are likely to counteract the irresponsiveness of *SOC1* to FT, and the absence of difference between the unsteady states leading to the inflorescence and floral identities observed in our first exploration. However, this will increase even further the number of possible models.

Constraining the search space of Boolean models with a defined network topology greatly reduces the number of models to explore. The exact figures depend on the topology. The Boolean network model formalism dictates that the state of any internal node is only dependent on the states of its regulators. Therefore, if node *i* has *r*_*i*_ regulators, its truth table will have $2^{r_{i}}$ entries. As a consequence, there are $2^{2^{r_{i}}}$ ways of choosing the truth table of node *i*. Building the whole model is equivalent to picking a combination of truth tables for all nodes, so the number of models in the search space is given in Eq 1.

$$\overset{n}{\underset{i=1}{\prod }}2^{2^{r_{i}}}=2^{\sum_{i=1}^{n}2^{r_{i}}}$$ (1)

With the topology from Fornara *et al*. plus the two extra interactions, $\sum_{i=1}^{n}2^{r_{i}}$ equals 54.

**Fig 3:**
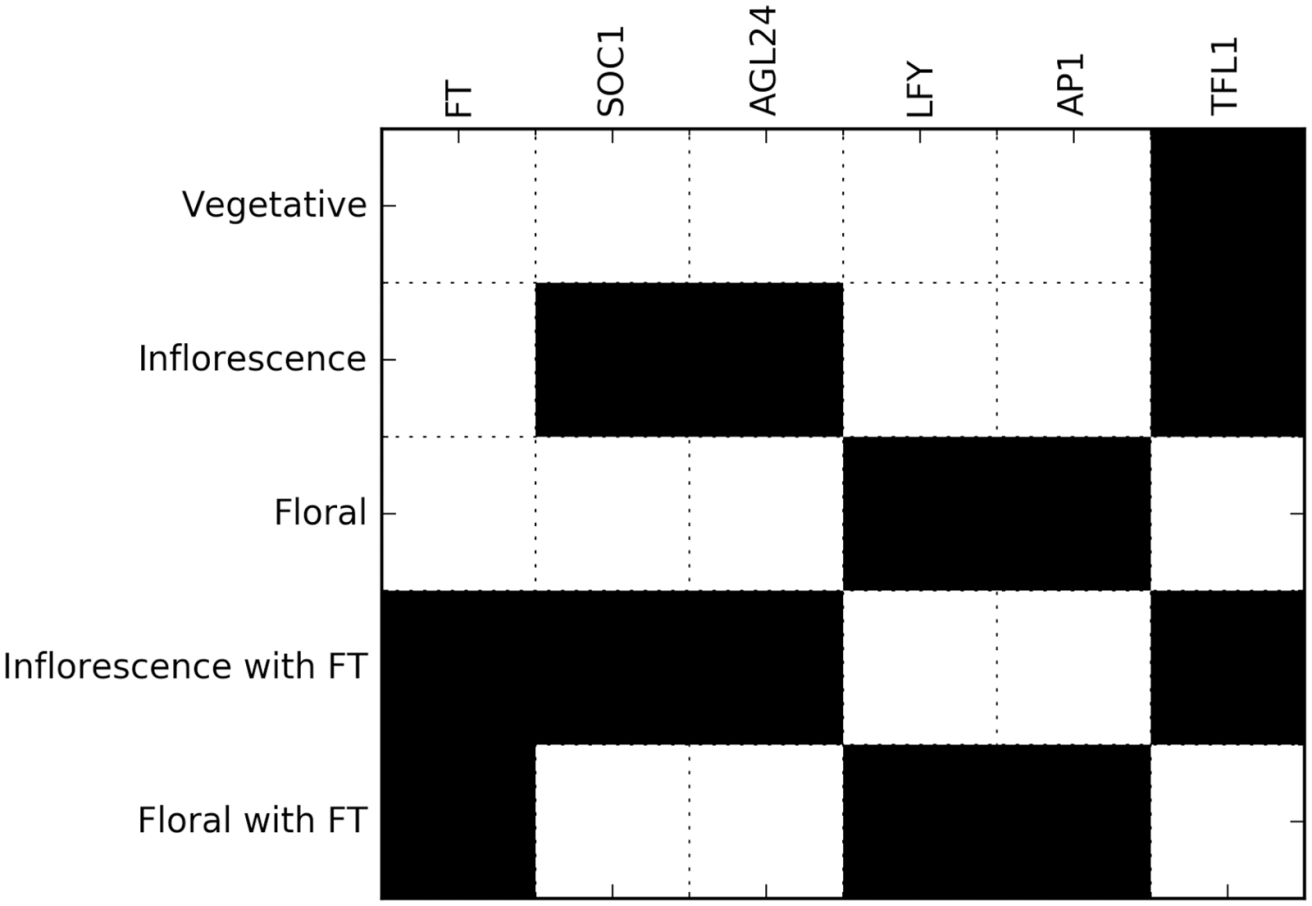
Required steady states for the exhaustive search. Each row corresponds to a desired steady state, and each column to a gene. Black and white cells respectively indicate whether a gene is expressed or not.

**Fig 4:**
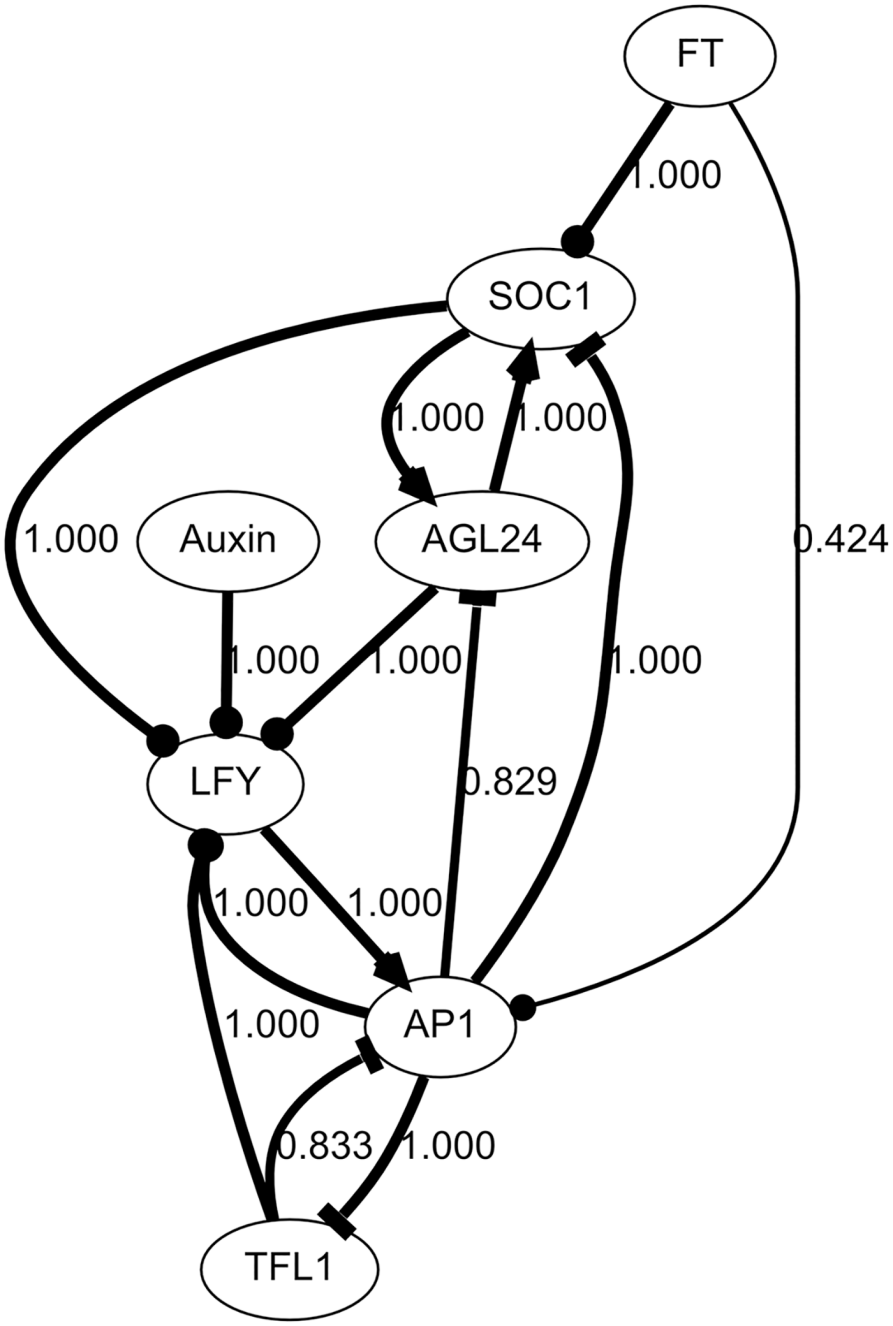
Aggregate graph of the models generated by exhaustive search on Fornara data set with 2 extra interactions. The nodes of the graph represent the species of the regulatory network, which are also nodes of the Boolean network models. Edges represent regulatory interactions between regulators and their targets. Arrowheads are placed on the side of the target species. V-, T- and O-shaped arrowheads respectively denote up-regulation, down-regulation, and interactions that can fall in either category, depending on the context and the model. Edge thicknesses and edge labels indicate the frequency of occurrence of the associated interactions, across all the models generated. Owing to the very large number of models obtained, a frequency displayed as 1.000 does not necessarily mean all models.

As a consequence, there are 2^54^ models in the search space after excluding models that do not conform to prior knowledge (down from 2^320^). Details of the calculation are provided in Table 2. Furthermore, most of them can be ruled out because they are not compatible with the observed steady states (see S2 Text). In this case, only 2^37^ solutions presented the required steady states (Fig 3). As evidenced by the formulae, adding new interactions becomes more and more expensive. In particular, the latest two interactions added into the data set, AP1→SOC1 and Auxin→LFY, increased the size of the search space 2^4^-fold and 2^16^-fold, respectively. This brought the problem close to the limit of what was computationally feasible. Performing the exhaustive search on this problem takes about 1.5 years with current CPUs, but was achieved using a 192-core High-Performance-Computing cluster running for 3 days. The search returned 1.6 billion suitable models. These solutions were used to build an aggregate topology graph of the GRN (Fig 4), using the methods described in S6 Text.

**Table 2:** Contributions of each gene to the number of models to explore.

*i*	*r*_*i*_	$2^{r_{i}}$
*SOC1*	3	8
*AGL24*	2	4
*LFY*	5	32
*AP1*	3	8
*TFL1*	1	2

The 1.6 billion models represent networks with mostly similar topologies. Among the 14 interactions allowed in the search space, all appear in at least some models, and 11 appear in all models. 7 interactions can clearly be labelled as positive or negative, but the other 7 remain ambiguous. This happens because either an interaction is sometimes positive and negative in the same model, depending on which other regulators are present, or it is positive in some models and negative in others.

**Fig 5:**
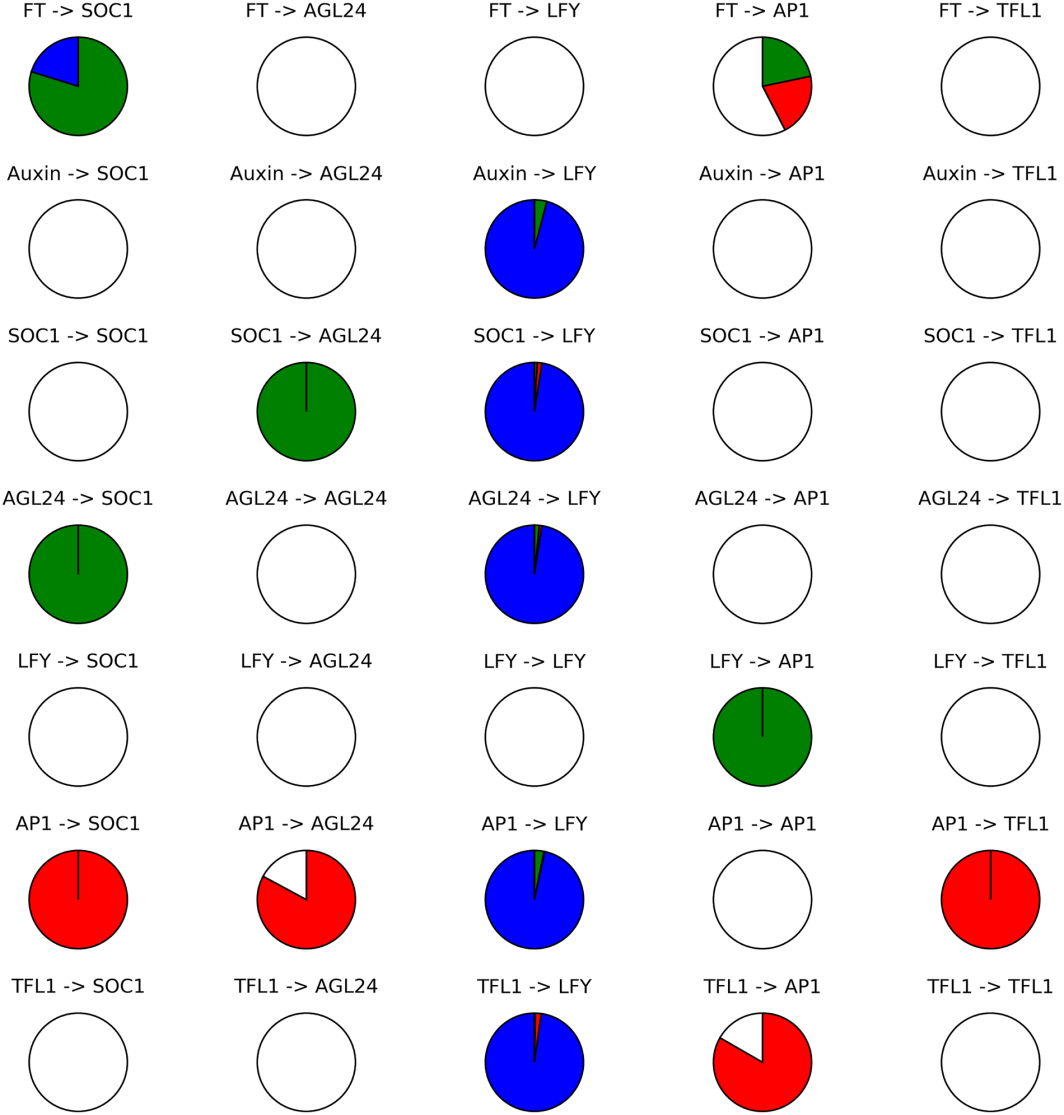
Distribution of interaction types per interaction across the set of models generated by exhaustive search. Each pie chart indicates the proportions of models in which the associated interaction is positive (green), negative (red), ambivalent (blue) or non-existent (white). Thus, FT was never seen to influence *LFY*, but it did activate or repress *AP1* (as shown by the red and green segments) in ~17% each of the models found.

Fig 5 shows the proportions of models in which each interaction is positive, negative, ambivalent, and absent. In most models, the interactions controlling *LFY* are ambivalent, meaning that the regulators of *LFY* can be both activators and repressors, depending on the combination of other regulators. Such behaviors do not seem very plausible. Instead, it is likely that these models are simply artefacts resulting from the high number of regulators of *LFY* and the comparatively small amount of information about the behavior of *LFY*: many combinations of *LFY* regulators are possible, but the actual behavior of *LFY* is unknown in most of them.

### A higher resolution description of gene expression during the transition can be established from in situ hybridization (ISH) data

**Fig 6:**
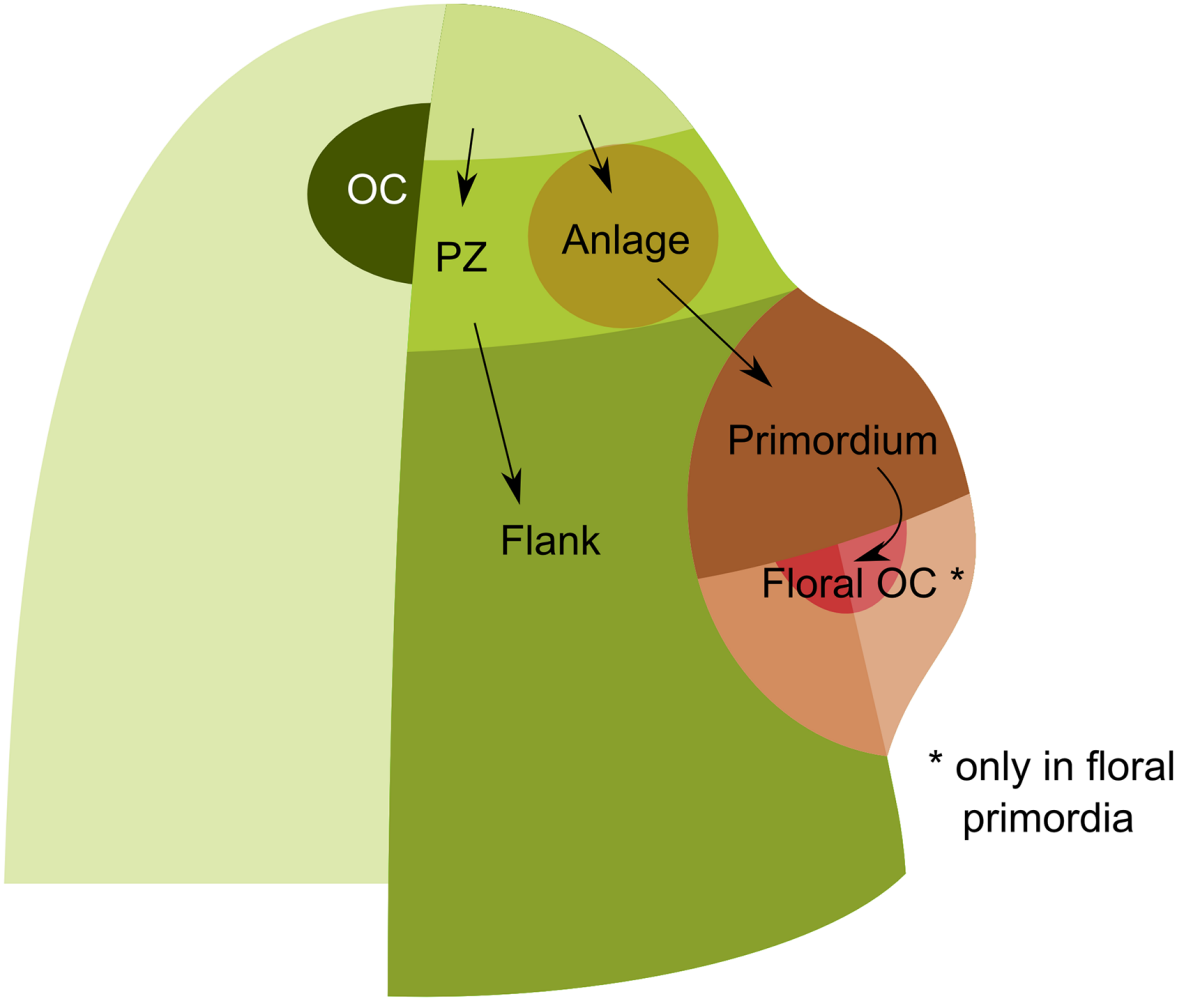
SAM domains. A Shoot Apical Meristem is a dome-shaped structure whose zonal cell types (identities) have been depicted in different colors. The Organizing Center (OC), Flank, Peripheral Zone (PZ), Central Zone (CZ) and internal cells are shown in progressively paler shades of green, while an Anlage and Primordium are shown in two shades of brown. Cells in the CZ grow, divide and differentiate into the PZ or an Anlage, which respectively go on to become the flank or a primordium. Each Anlage is generated periodically. In a vegetative meristem, these Anlagen go on to produce primordia that lack their own OC and grow into leaves. Inflorescence meristems produce Anlagen that become floral primordia that do contain their own OCs (colored red).

**Fig 7:**
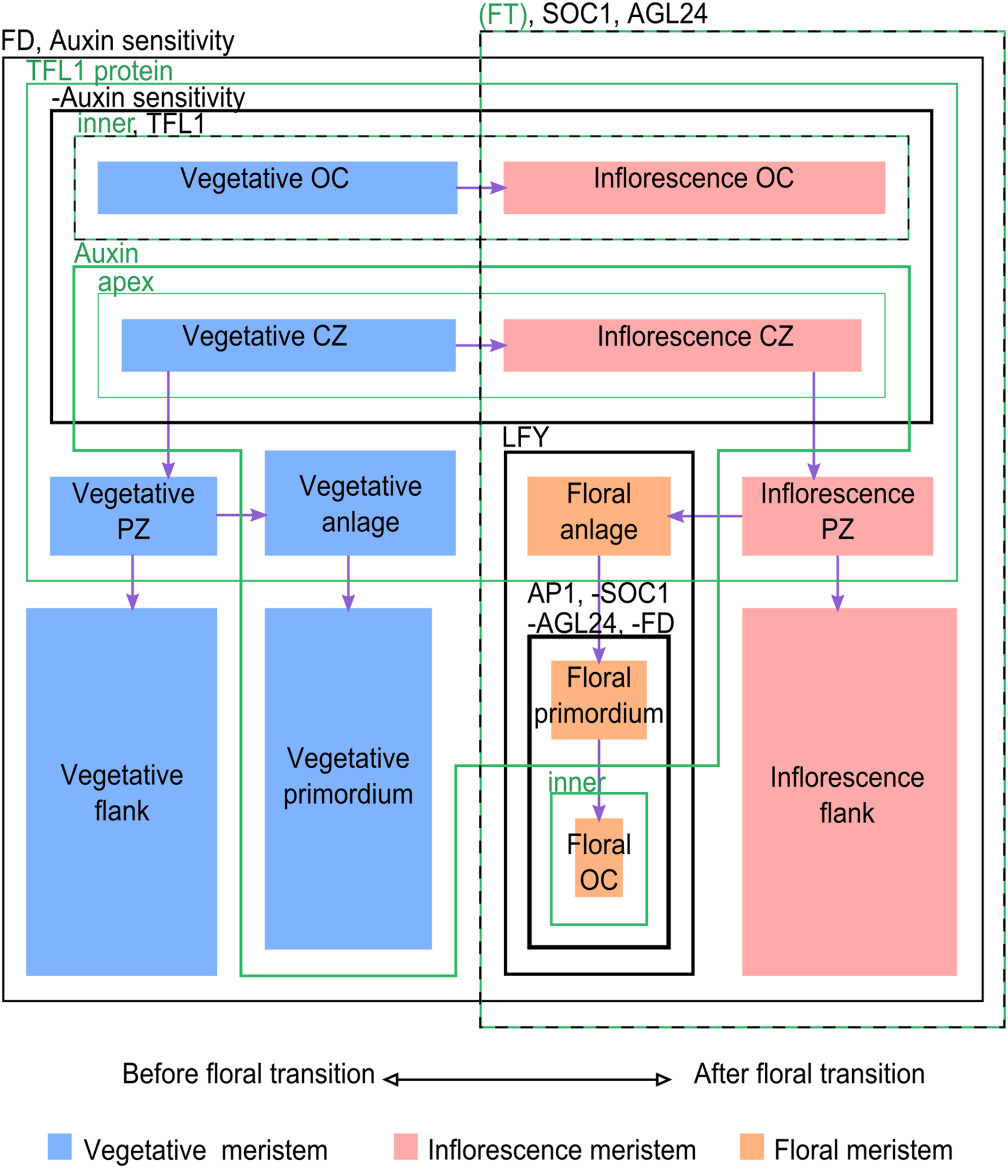
SAM gene expression domains in time and space. Distribution of gene-expression patterns. Green and black contours mark the expression domains of the species mentioned in the upper left corners of the boxes. A minus sign before a gene name means the frame marks a hole in the expression domain of that gene. The green species are those used as triggers of the transitions between developmental stages. Transitions (symbolized by purple arrows) are triggered by toggling the variables associated with the green species (i.e. crossing green lines on the diagram), which pushes the system towards a new identity, often causing black species to also toggle their values (i.e. cross black lines on the diagram). Identities are represented as colored areas for clarity, the surface of these areas is not representative. The left-hand and the right-hand halves of the picture are temporally separate, all other separations are spatial.

A survey has been carried out of published ISH studies of *AGL24*, *AP1*, *LFY*, *SOC1*, *TFL1* and *FD*, with which FT interacts (see S1 Table). As well as the three classical meristematic identities (Table 1), this survey has revealed additional identities, most of them matching zones already characterized in studies of SAM development [23], see Figs 2, 6 and 7. Unless otherwise stated, all of these identities have variants in both the vegetative and floral phases.

The first identity is the organizing center (OC). It is classically defined as the expression domain of *WUS*, but it also seems to express *TFL1* [24,25], another mobile protein that is transported towards the apex. The second identity is the central zone (CZ), which contains stem cells and is located at the very apex of the meristem. These cells are unable to initiate the formation of a primordium in response to auxin [26], possibly because their auxin sensitivity has been disrupted, as suggested by the expression patterns of some genes of the *ARF* family [27]. The third is the peripheral zone (PZ), vegetative or inflorescence, which surrounds the CZ. We define its border as that of the diffusion domain of the TFL1 protein [24]. Within the PZ, some cells actually belong to another (fourth) identity: anlagen, which are founder cells of lateral organs. Their defining characteristic is a high concentration of auxin. Floral anlagen start expressing *LFY* [28]. The fifth identity is the primordia for anlagen that have gone through the boundary of the TFL1 protein domain, which express *AP1* [29,30], but not *FD* [29], *SOC1* [30] or *AGL24* [31]. Finally, the sixth is the meristem flank, which surrounds primordia. Compared to the peripheral zone, its differences are that it does not have TFL1 proteins [24] and it is insensitive to auxin treatment [32].

In addition to these known steady states, knowledge of the processes involved in plant development has enabled us to generate a list of initial steady states, perturbations and resulting steady states (Table 3). These steady states and transitions were also complemented with information inferred from the phenotypes of the *tfl1* and the *ap1* mutants (see Table 4). Studying the *ap1* mutant led us to consider a seventh zone: the floral OC, which does not have any counterpart in the vegetative SAM. In WT plants, it is very similar to the floral primordium, except that it is located deeper within the meristem, and we assume it does not have a high concentration of auxin. In the *ap1* mutant, this territory is expected to turn into an inflorescence OC instead, paving the way for a recursive, cauliflower-like inflorescence architecture.

**Table 3:** Developmental transformations in WT.

Initial steady state	Perturbation	Final steady state
Vegetative CZ	- apex - auxin	Vegetative PZ
Vegetative PZ	- TFL1 protein	Vegetative flank
Vegetative PZ	+ auxin	Vegetative anlagen
Vegetative anlagen	- TFL1 protein	Vegetative primordium
Inflorescence CZ	- apex - auxin	Inflorescence PZ
Inflorescence PZ	- TFL1 protein	Inflorescence flank
Inflorescence PZ	+ auxin	Floral anlagen
Floral anlagen	- TFL1 protein	Floral primordium
Floral primordium	+ inner - auxin	Floral OC
Vegetative OC	+ FT	Inflorescence OC
Vegetative CZ	+ FT	Inflorescence CZ
Inflorescence CZ with FT	- apex - auxin	Inflorescence PZ with FT
Inflorescence PZ with FT	- TFL1 protein	Inflorescence flank with FT
Inflorescence PZ with FT	+ auxin	Floral anlage with FT
Floral anlage with FT	- TFL1 protein	Floral primordium with FT
Floral primordium with FT	+ inner - auxin	Floral OC with FT

**Table 4:** Transitions in mutant plants.

Mutation	Initial steady state	Perturbation	Resulting steady state
*tfl1*	Vegetative CZ	+ FT	A state with *AP1*
*ap1*	Floral anlagen	- TFL1 protein	Floral primordium in *ap1*
*ap1*	Floral primordium in *ap1*	+ inner - auxin	Inflorescence OC (similar to WT)
*ap1*	Floral primordium in *ap1*	+ apex + TFL1 protein	Inflorescence CZ (similar to WT)

The additional data provided by ISH was unfortunately shown by exhaustive search to be incompatible with the supplemented Fornara topology, as some of the observed steady states (Fig 2) provide conflicting information about the regulation of some genes, implying that the topology is incomplete. As a consequence, in order to solve this problem, it was crucial to develop a method that can suggest new regulatory edges for the network. One approach involves the use of Genetic Programming (GP). There are two motives for developing such an approach: the need for simpler over complex/implausible regulatory interactions, and a non-exhaustive strategy of exploration of the search space should be both more cost-effective and allow the solving of complex cases that involve more species and interactions. This performance gain can also be used to explore models that do not perfectly match prior knowledge, and hence potentially identify previously unknown interactions.

### A genetic programming algorithm proposes Boolean models that explain the meristem development during the transition

**Fig 8:**
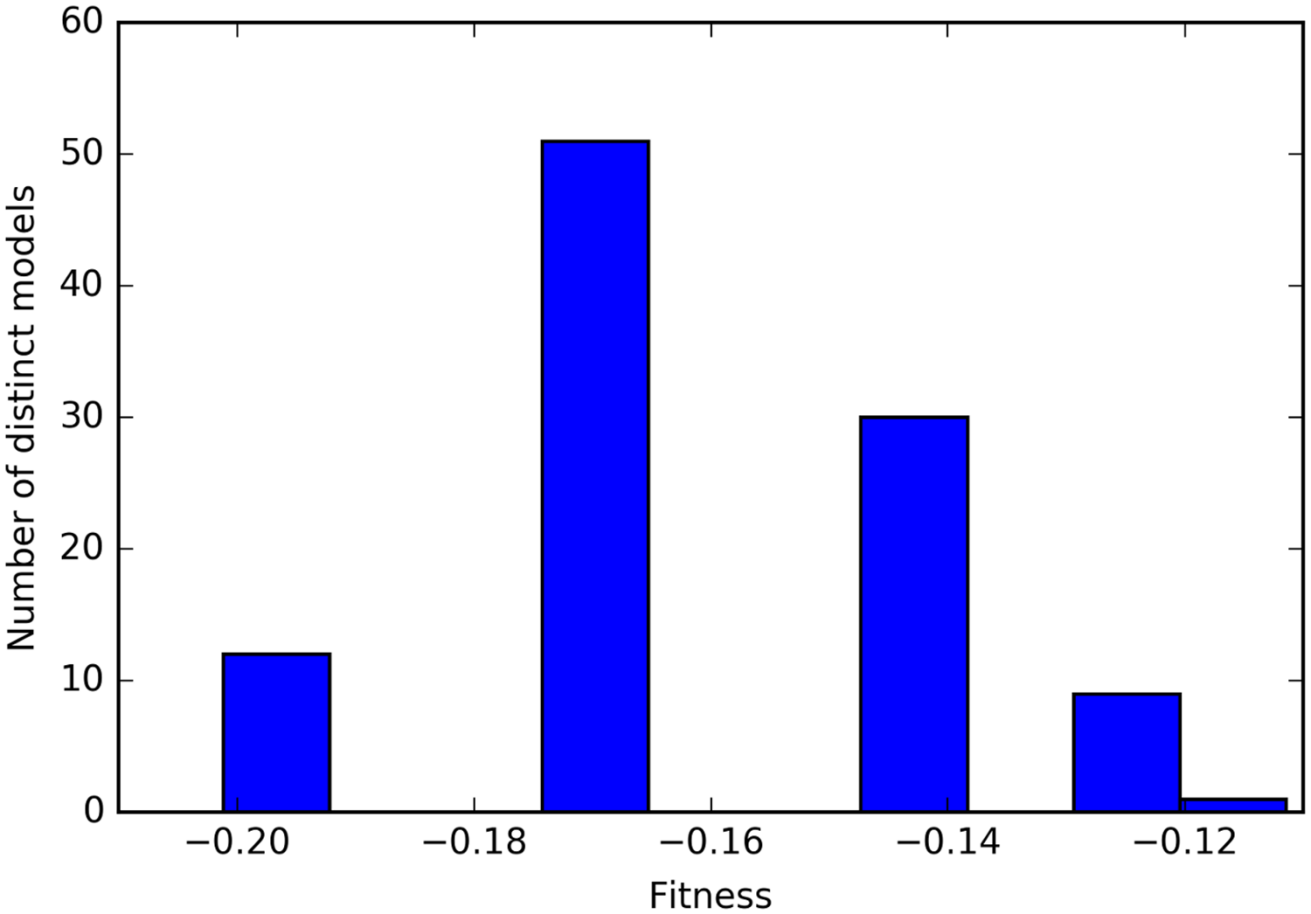
Distribution of the fitness values of the 103 distinct models generated by genetic programming. The formula for this can be found in S5 Text. Models found by genetic programming spontaneously segregate into clusters corresponding to their fitness values. Each cluster corresponds to a different number of novel interactions introduced into the regulation network. The algorithm attempts to find models with the fewest novel interactions possible, i.e. those with the lowest fitness values. It does however not always succeed in finding models with the actual lowest possible number of novel interactions, hence the presence of several clusters on the diagram.

**Fig 9:**
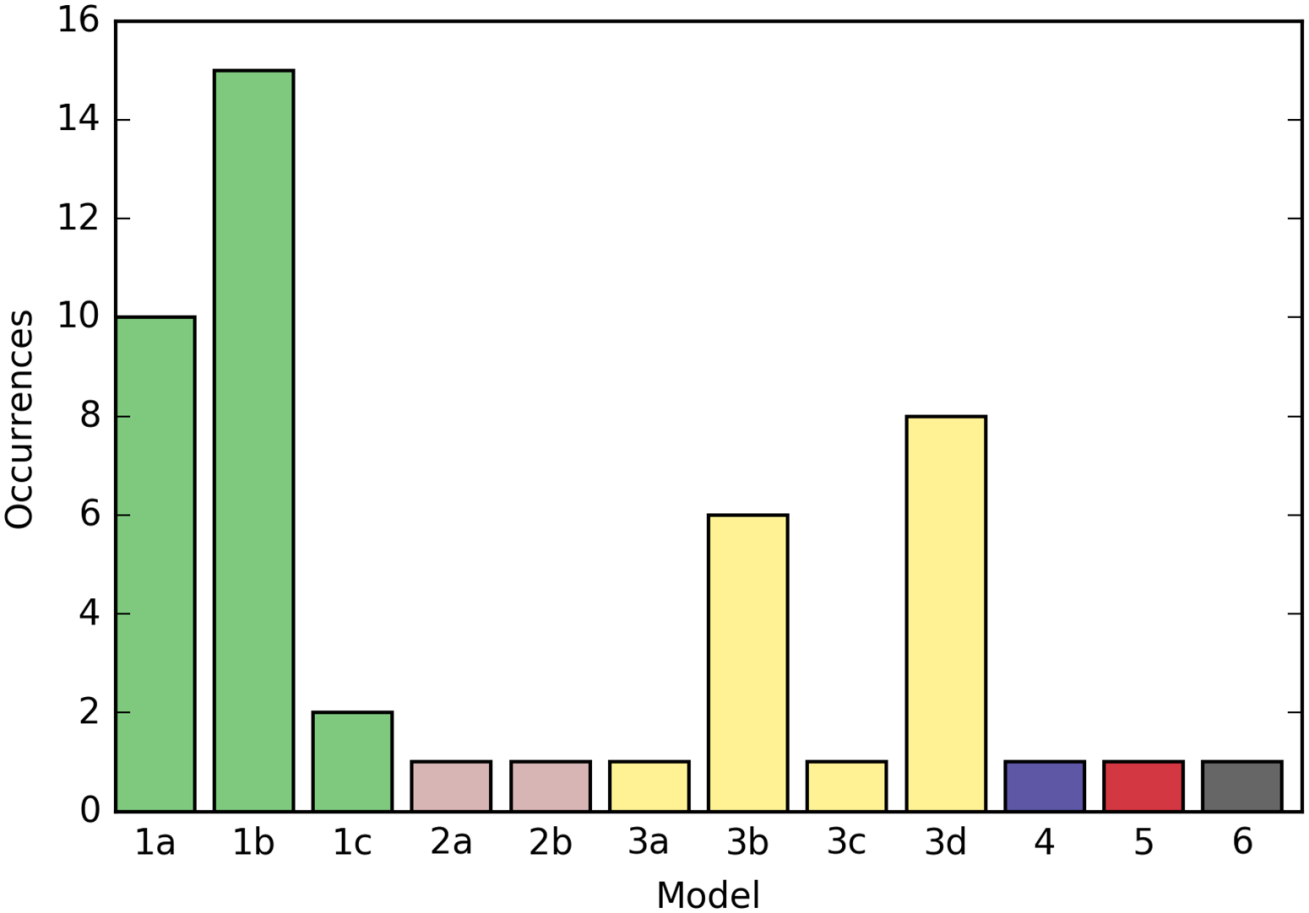
Counts of the distinct models generated by the GP algorithm, with fitness < −0.18. Models with the same number have the same fitness value. The approach favors models with lower fitness values, but even at a given fitness value (1a-1c, 3a-3d), not all models are found with the same frequency, suggesting that some may be easier to find than others.

465 models fitting the observations were generated using this approach. As these results included models that shared the same truth table, they could be filtered down to 103 distinct models (i.e. models with distinct truth tables). These models can be clearly classified according to their fitness values (Fig 8; lower is better). The presence of clearly separated peaks is due to the way the fitness function was constructed. Each peak represents a different number of novel interactions. The number of copies per distinct model from the first peak (fitness < -0.18) is plotted in Fig 9. It empirically shows that not all models of approximately equal fitness will be found with similar frequencies by the algorithm, and this also applies when all the models were considered (see S2 Fig).

**Fig 10:**
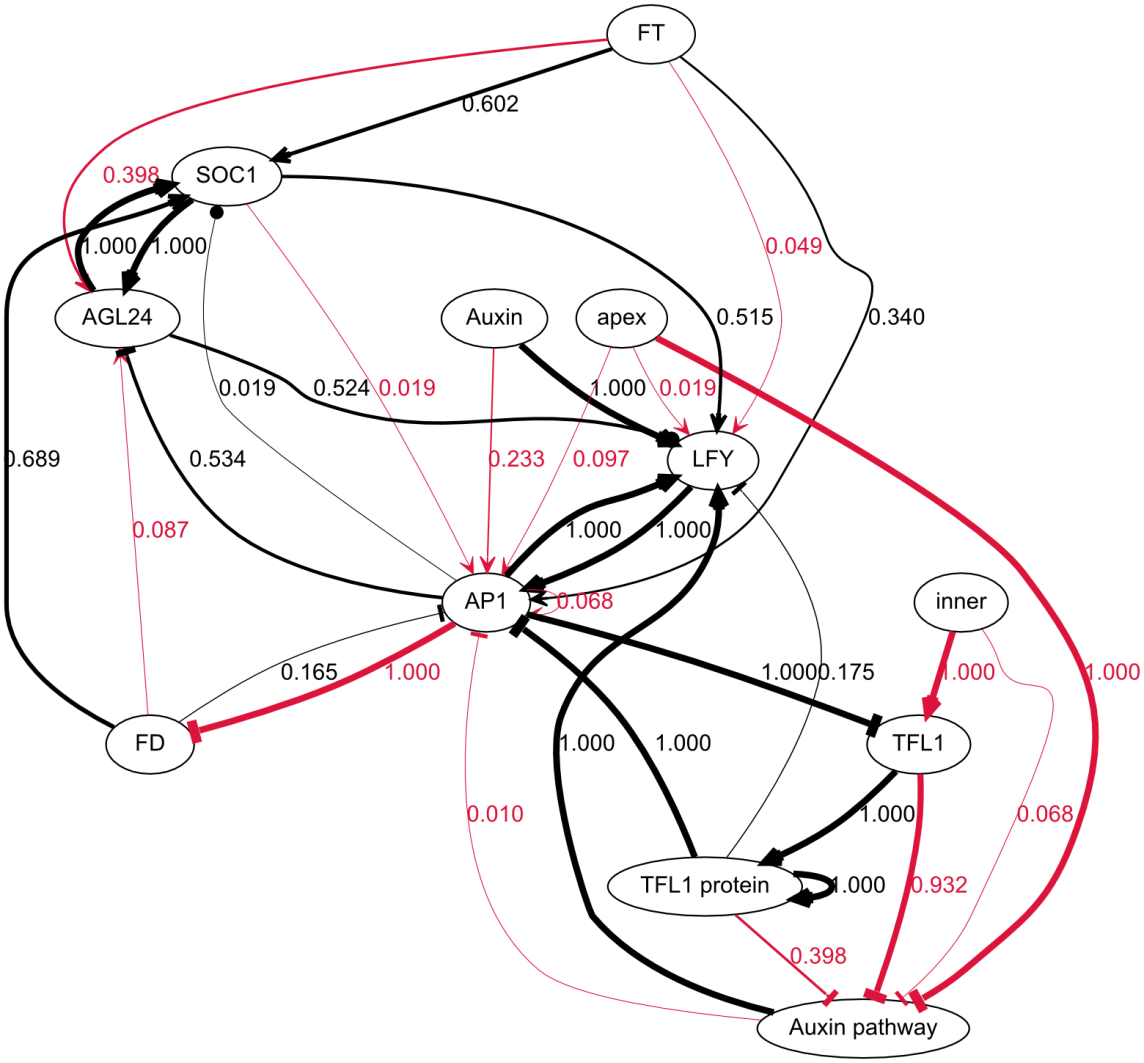
Interactions found in the 103 networks generated by genetic programming. Black and red edges are respectively part of the prior knowledge and novel. Edge labels represent the frequencies of their respective edges. Many novel interactions appear in at least some of the 103 models, but most of them with low frequencies. The interactions involving apex and inner however both have frequencies of 1. This confirms that the variables apex and inner, as they were defined, would be able to explain the patterning of the auxin signaling pathway and *TFL1*, respectively, although additional work would be needed to explain how apex and inner can be defined molecularly. This also shows that no way to substitute apex or inner with other variables could be found, unless it would involve substantially more novel interactions.

As mentioned previously, the topology provided to the algorithm did not allow, as is, for any solutions to be found. As a consequence, all solutions proposed by GP involve additional interactions that were not part of the prior knowledge. An aggregate graph of the topologies of the 103 models is presented in Fig 10. It reveals numerous potential novel interactions, many of which occur at low frequencies (< 10%). This is because the set includes sub-optimal models, as far as the parsimony of new interactions is concerned (i.e. they include models that have more novel interactions than necessary). This can be addressed by retaining only the models with lower (i.e. better) fitness values.

**Fig 11:**
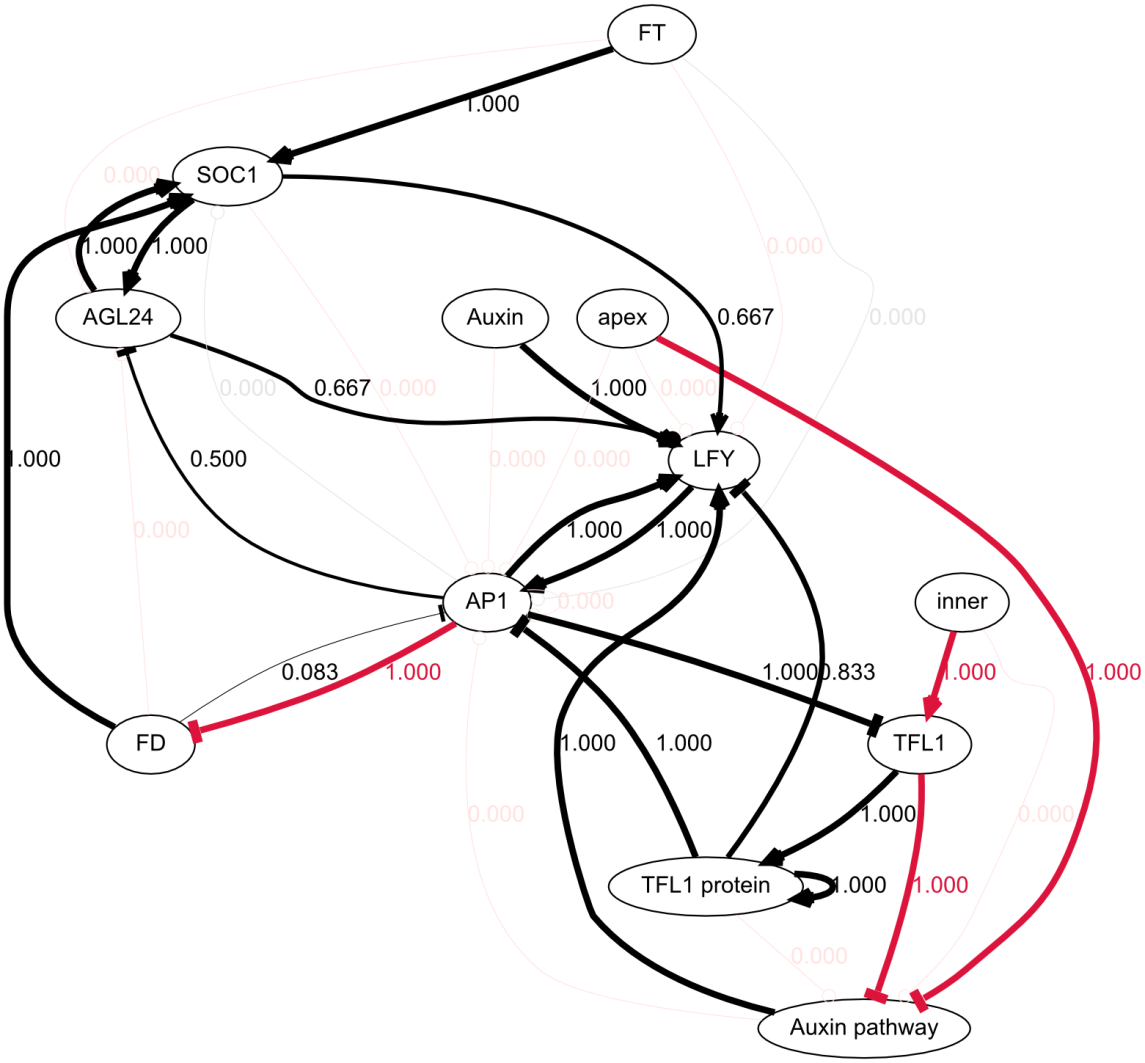
Interactions found in the 12 networks in the first peak of fitness. The naming and color scheme are as described for Fig 9. This shows the repressions of *FD* by *AP1* and of the auxin pathway by *TFL1* are the most straightforward additions required to make the network consistent with the data. This also shows that some interactions are not required to explain the data, namely *FT* → *AP1*, *FD* → *AP1* and *AP1* → *SOC1*.

In the following, only the best models (fitness values < -0.18) were retained, as they are—by construction—the models with the fewest novel interactions (4 in total). Some are more parsimonious than others in terms of known interactions (see section S2 Table), but we will consider them equally relevant here, as our main focus is the study of minimal sets of novel interactions able to complement published networks. The aggregate graph of this selection is presented in Fig 11. The 12 models selected this way suggest:

- *FD* is repressed by *AP1*; this would constitute a negative feedback loop, whereby *FD* activates floral identity genes before indirectly turning itself off;
- *SOC1* is not necessarily repressed directly by *AP1*; the results of the exhaustive search had shown that a negative feedback loop was necessary, but it might be the same as that of *FD*;
- *AP1* is not necessarily activated directly by FT; an indirect activation pathway through *SOC1* and *LFY* is sufficient;
- *TFL1* is upregulated by a non-modelled factor present in the inner tissue of the meristem, or a modelled factor with unknown interactions occurring in the inner tissue of the meristem;
- The auxin pathway is disrupted by *TFL1* and a non-modelled factor present in the CZ, or a modelled factor with unknown interactions occurring in the CZ of the meristem.

**Fig 12:**
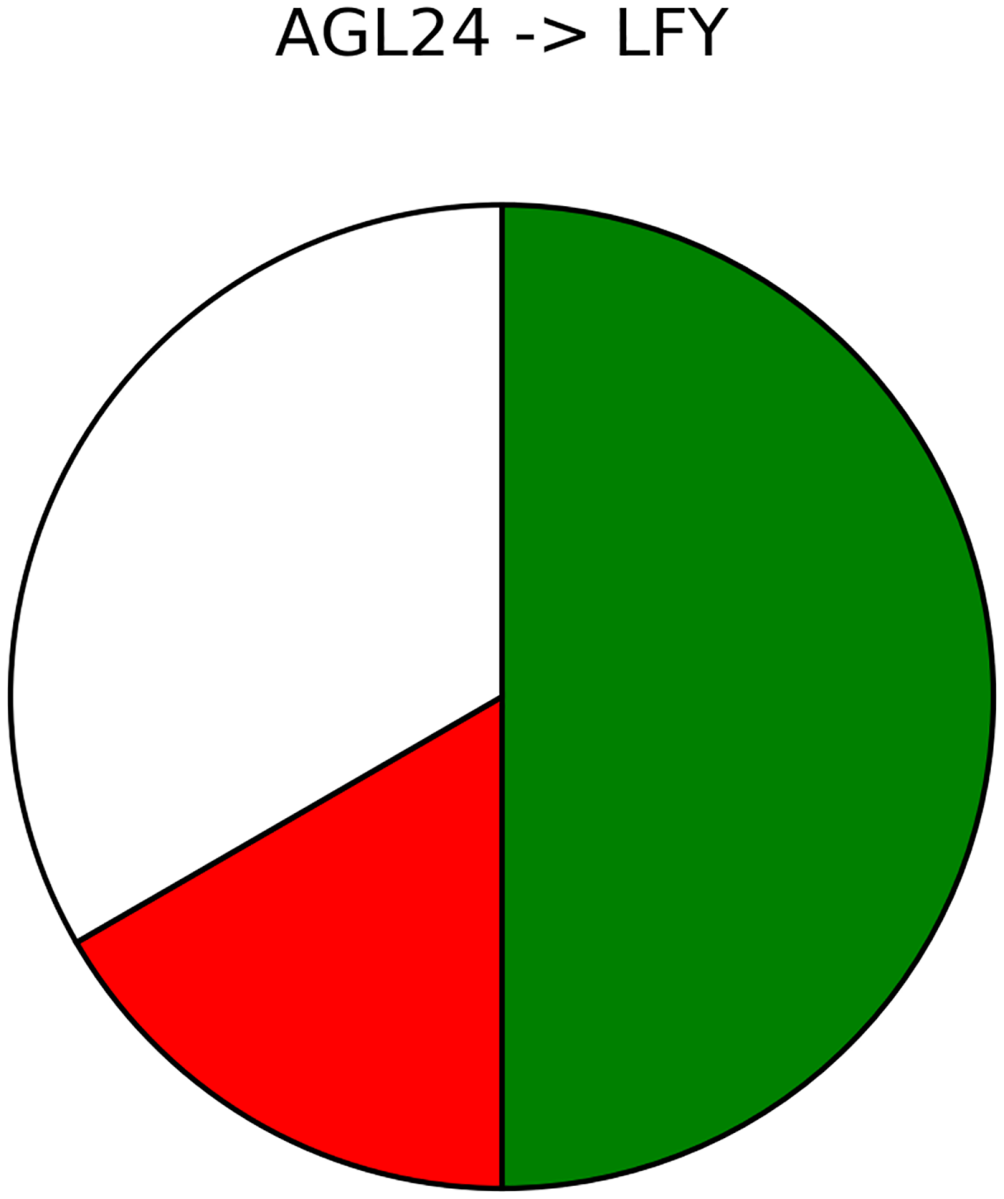
The *AGL24* → *LFY* interaction types across the subset of 12 models. The pie chart indicates the proportion of models in which the associated interaction is positive (green), negative (red), or non-existent (white).

In this subset of solutions, only one interaction (*AGL24* → *LFY*) is of undefined nature in the aggregate of the 12 models. This interaction is however never undefined within any given model (Fig 12), instead there are some models where it is positive, and some where it is negative. This shows that this GP approach is able to avoid complex models. The equations of the 12 models are given in S2 Table. Among these 12 distinct models, 5 interactions are not present in all models:

- TFL1 protein → *LFY*;
- *FD* → *AP1*;
- *SOC1* → *LFY*;
- *AGL24* → *LFY*;
- *AP1* → *AGL24*.

Principal component analysis (PCA) was carried out to determine the number of degrees of freedom in the set of 12 models. It showed this set was truly 5-dimensional. 91% of variance can be explained by the first three components (Table 5). The first component only covers interactions *SOC1* → *LFY* and *AGL24* → *LFY*, with opposite coefficients, showing the *SOC1* and *AGL24* nodes can play similar roles in the regulation of *LFY* in the generated models. The second component is mostly composed of AP1 → AGL24, probably because it is not necessary for a model to fit the observations: the most concise models generated do not include that interaction at all (see S2 Table).

**Table 5:** Principal components of the variability in the subset of 12 models.

Principal Component	TFL1 protein → *LFY*	*FD* → *AP1*	*SOC1* → *LFY*	*AGL24* → *LFY*	*AP1* → *AGL24*	Percentage of variance explained
#1	-0.000	0.000	0.707	- 0.707	0.000	0.366
#2	-0.357	0.362	0.190	0.190	0.819	0.295
#3	0.622	-0.259	-0.350	-0.350	0.548	0.253

The numbers in the interaction columns are the components of the PCA vector, the more extreme (+/-) the value, the more it is contributing to the associated component of variance.

Looking at combinations of interactions model per model provides additional insight. Noticeably, *LFY* is always upregulated by *SOC1*, *AGL24* or both, in each of the proposed solutions (Table 6). It highlights the importance of an activation path from inflorescence genes (*SOC1* and *AGL24*) to the floral identity gene *LFY*, and confirms that one such path is theoretically sufficient. However, if only one of them activates *LFY*, the algorithm is not able to suggest which one from the available data.

**Table 6:** Combinations of interaction types in the best cluster of GP-generated models.

TFL1 protein → *LFY*	*FD* → *AP1*	*SOC1* → *LFY*	*AGL24* → *LFY*	*AP1* → *AGL24*	Counts
-1	0	1	0	0	2
-1	0	0	1	-1	2
-1	0	0	1	0	2
-1	0	1	0	-1	2
0	-1	1	-1	-1	1
-1	0	1	1	0	1
0	0	1	-1	0	1
-1	0	1	1	-1	1

Each row corresponds to the set of interactions (-1, 1 and 0 denoting inhibition, activation and no interaction) with the final column showing the number of models in which this set was found.

Interestingly, none of the configurations reported in Table 6 involves all five interactions, even though they do all feature in the topology reviewed by Fornara *et al*. However, the missing interactions can be either of the five. This shows there is not only redundancy between *SOC1* and *AGL24*, but also at a higher level.

## Discussion

In this paper, we have described a succession of approaches aiming to build Boolean models able to reproduce a set of spatio-temporal gene expression patterns, whilst complying with prior knowledge on the regulatory topology. Starting from a brute force approach exhaustively enumerating a list of candidate models, we have been led to more sophisticated developments based on genetic programming. It seems likely that other systems involving cell differentiation and tissue patterning would require similar refinements, but it might be, in cases where prior biological knowledge is detailed enough, that the simplest approach leads to relevant conclusions. Therefore, the results have included all the different steps with some details, as summarized now.

The most naïve search strategy, exhaustive search, can only be carried out on very simple models, though it can be improved upon by restricting the search space to models conforming to a predetermined network topology. This drastically simplifies the problem, however, it might still not be enough if the network topology is too complex. Another issue is that it requires a sufficiently comprehensive network topology, which might not be available. However, even if these two problems do not arise, solutions generated this way may not be satisfying, as they are likely to involve complex, unlikely regulatory mechanisms.

These three problems are addressed by the GP approach used here. Genetic algorithms are known to be efficient at exploring high-dimensional spaces, such as the space of all Boolean models involving a set of nodes. Genetic programming has the added benefit of being able to generate Boolean equations directly, which makes it easier to target models involving simpler, more plausible regulatory interactions. This approach has successfully been applied to the regulatory network controlling cell identity in the SAM, resulting on the formulation of several plausible models and the suggestion of novel regulatory interactions absent from the starting network topology, but confirmed by independent laboratory work.

A large part of the input data in this work has been extracted from *in situ* hybridization experiments. This shows the locations the mRNA of the studied genes, but not their proteins, which is an issue for mobile proteins, such as FT and TFL1. Although the greatest care was taken when interpreting ISH pictures, comparing plants of different ages at different times, and grown in different conditions may be a source of errors. Confocal imaging of multiple fluorescent fusion proteins could help with both matters, as it provides a way of tracing proteins and studying how they co-localize. Following the development of the same plant through time is also possible with this technique, but it would take many months to generate such plant lines.

### Lack of mutant data

The core of our approach is based on the use of ISH data. Unfortunately, this kind of data is usually not available for mutants. This has consequences for the models that can be generated. Indeed, real biological regulatory networks are usually robust to mutations, as regulators are often encoded by a family of related genes, providing redundancy. However, our GP approach generates models as simple as possible, and there are few data about expression profiles in mutants, so it has no reason to try to represent the robustness of the real network. This means the algorithm will build models featuring little, if any, redundancy.

### Applicability of the method

This method, based on co-expression profiles and genetic programming, has been successfully applied to the case of the network controlling cell identity in the SAM. Although it has not been tested on other biological networks, it should be applicable to other networks when appropriate data sets are available. It would be interesting to see how well the method performs on other cases, and, in particular, if the trade-off between computation time and quality of the output models is satisfactory across all cases. It is entirely possible that this trade-off could be improved using a different set of parameters for the GP algorithm, both as default values and as problem-specific values. This is because little optimization has been carried out in this area, due to the high computational cost associated with it.

### Role of *AP1* and *TFL1*

This work suggests that *AP1* represses *FD*. While this was not reported by Liu *et al*. or Fornara *et al*., it has since been published [33]. The GP output also suggested *AP1* does not necessarily need to directly down-regulate *SOC1*, as this would be redundant with an indirect repression via *FD*. This might be tested experimentally in an *FD*-overexpressing plant. If *SOC1* is not down-regulated in floral primordia, it would confirm that the repression of *SOC1* by *AP1* goes through *FD*. Alternatively, it is possible that both regulatory features occur and this is a case of feed-forward repression.

One of the aims of this work is to investigate the place of *TFL1* in the regulation of cell identity in the SAM. To make this possible, variables inner and apex were introduced for the following reasons. First, very little is known about the regulation of *TFL1*, which makes it difficult to produce models where *TFL1* is expressed in the right conditions. The patterning of *TFL1* is, however, very similar to that of *WUS*, for which a patterning mechanism combining inhibition in outer tissues and sensitivity to activation in inner tissues has been proposed [30]. An “inner” node was added to the network to enable similar models for *TFL1*. Second, *TFL1* seems to affect the identity of CZs. Indeed, floral meristems, which are usually determinate, instead generate recursive cauliflower-like patterns in the *ap1*/*cal* mutants, where *TFL1* is expressed ectopically. Conversely, the SAM becomes determinate in *tfl1* mutants, as the meristem turns into a flower after the transition. Since the apices of the SAM and floral meristems appear to have similar behaviors in some genetic backgrounds, we postulated that those apices share some unknown properties responsible for this shared behavior, and introduced a variable called “apex” accordingly.

It is not clear which molecular species correspond to the spatial information implied by variables inner and apex, but some genes exhibit the relevant expression patterns. Inner seems to correlate with *AHK4* [34] and apex to *CLV3* [35]. Interestingly, these two genes are involved in the *WUSCHEL*-*CLAVATA* negative feedback loop. As *WUS* and *TFL1* share similar expression patterns and their expression levels are correlated, it seems likely that *TFL1* and genes of this loop are somehow connected. Should it not be the case, the patterns of *AHK4* and *CLV3* still prove that genes with patterns appropriate to explain those of inner and apex do exist.

### Extension to quantitative modelling

Inferring a quantitative model of the network controlling SAM identity—such as an ODE or PDE model—by genetic programming might be possible. The major challenges, however, are that it would add a parameter optimization problem for each system of equations to assess, and the simulations of ODE models are more expensive than those of Boolean models. However, instead of trying to infer a quantitative model directly, another approach could be to convert the Boolean models into ODE models using predefined methods [36,37]. These quantitative models could then be simulated in a spatially explicit context, such as a 3D tissue mesh, which would enable the simulation of transitions in a more explicit way (growth, cell division, diffusion, transport). The main limitation of such developments is the lack of any nondestructive experimental method to measure quantitatively the gene expression patterns of cells *in situ* in organs, so that the quantitative outputs of differential models would have no experimental counterpart for comparison.

## Materials and methods

### Data

#### Classical 3-identity model

There are traditionally three characterized identities for cells constituting the SAM: vegetative, inflorescence and floral [4]. Some genes are commonly considered as characteristic of these profiles (Table 1). The vegetative profile represents any cell of the vegetative (pre-transition) SAM, as they do not seem to differ in the expression of any of the considered genes. The inflorescence profile represents cells of the main shoot of the inflorescence meristem (i.e.: primordia are excluded). The floral profile represents cells of the floral primordia. FT is necessary to induce the shift from vegetative to inflorescence in the OC and CZ, but once the inflorescence identity of CZ cells is acquired, FT is no longer required (memory effect).

#### Developmental transformations

The development of the SAM is assumed to take place through the occurrence of perturbations making the system transition from one steady state to another (Table 3).

#### Mutant phenotypes

In *tfl1* mutants, a terminal flower develops at the apex of the meristem. Another interesting case is the *ap1*/*cal* double mutant. *CAL* is a close homolog of *AP1*. When both are knocked out, the inflorescence develops into a cauliflower shape, where meristem primordia turn into inflorescence meristems and recursively generate new primordia. This information is summarized in Table 4.

### Genetic programming

Three criteria come into play in the fitness function, listed below in order of priority.

1. *n*_*violated*_: sum of the XOR distances between the required end steady states and the end steady states reached by the model, for the species deemed relevant (lower is better, always 0 for solutions to the problem); For each (I, P, F, C, M) transition (see S3 Text), attractor(P(I)) is calculated. If the latter is a steady state, the distance between attractor(P(I)) and F is the number of non-zero values in (P(I) XOR F) AND C. Otherwise, if attractor(P(I)) is a cycle, the model is rejected and the distance is set to the number of non-zero values in C;
2. *n*_*interactions*_: number of novel (i.e. not present in the data, see details below) interactions in the model (lower is better). For efficiency reasons, this is based on the equations of the model rather than its truth table. A novel interaction *ij* is considered included in a model if and only if *i* appears in the equation of j and interaction *ij* is not in the prior knowledge.
3. *n*_*terms*_: number of terms in the equations (including operators, lower is better). It is given by the number of nodes in the tree of the model. In order to optimize the fitness function, genetic programming algorithms produce successive generations of offspring.

The formula of the fitness function is presented in Eq 2.

$$\begin{array}{c} n_{violated}-\frac{1}{1+n_{interactions}-\frac{1}{1+n_{terms}}} \end{array}$$ (2)

This function does not allow any kind of trade-off: criteria with lower ranks always have priority over those with higher ranks.

As genetic algorithms can potentially get stuck in local minima of fitness functions, the scheme devised here mitigates this issue by running the algorithm multiple times and introducing transition data both progressively and in a different order each time. Each run follows the following process:

1. Establish a dataset D of known transitions;
2. Create an empty dataset D’;
3. Pick a transition in D randomly, and move it into D’;
4. Run the genetic programming algorithm until a solution that does not violate any transition in D’ is found or the algorithm times out (i.e. no solutions could be found in a preset number of generations after the latest transition was added). Repeat from step 3 until D is empty and a solution compatible with D’ is found (unless a time-out occurs);
5. If such a solution is found, keep running the genetic programming algorithm for a fixed number of iterations to come up with a simplified form. Save the best individual as a solution.

Running this algorithm multiple times generates different solutions.

## Supporting information

S1 Fig Aggregate graph of the models generated by exhaustive search on the topology reported by Fornara and colleagues. Nodes are genes. Edges represent regulatory interactions. Edge labels and edge thicknesses denote the occurrence frequencies of the associated interactions. V-, T- and O-shaped arrowheads indicate positive, negative and ambiguous interactions, respectively.
(TIF)

S2 Fig Counts of all distinct models generated by the GP algorithm. Models with the same number have the same fitness value.
(PNG)

S1 Table List of the genes and time points extracted from in situ hybridization images, and their sources. Dates are expressed as days after germination (dag), days after induction (dai) or as developmental stages when no other information was available (vegetative, transition or inflorescence).
(PDF)

S2 Table Equations and graphs of the twelve best models from the genetic programming search. (PDF)

S1 Text Boolean modeling. (PDF)

S2 Text Exhaustive search. (PDF)

S3 Text Verifying if a model can explain a transition. (PDF)

S4 Text Modeling of mutants. (PDF)

S5 Text Genetic programming. (PDF)

S6 Text Model analysis. (PDF)
