# Supplementary figures and images for: The logic of the floral transition: Reverse-engineering the switch controlling the identity of lateral organs
Source: PLoS Comput Biol. 2017 Sep 20;13(9):e1005744. doi: 10.1371/journal.pcbi.1005744 (PMC5624648; doi:10.1371/journal.pcbi.1005744)

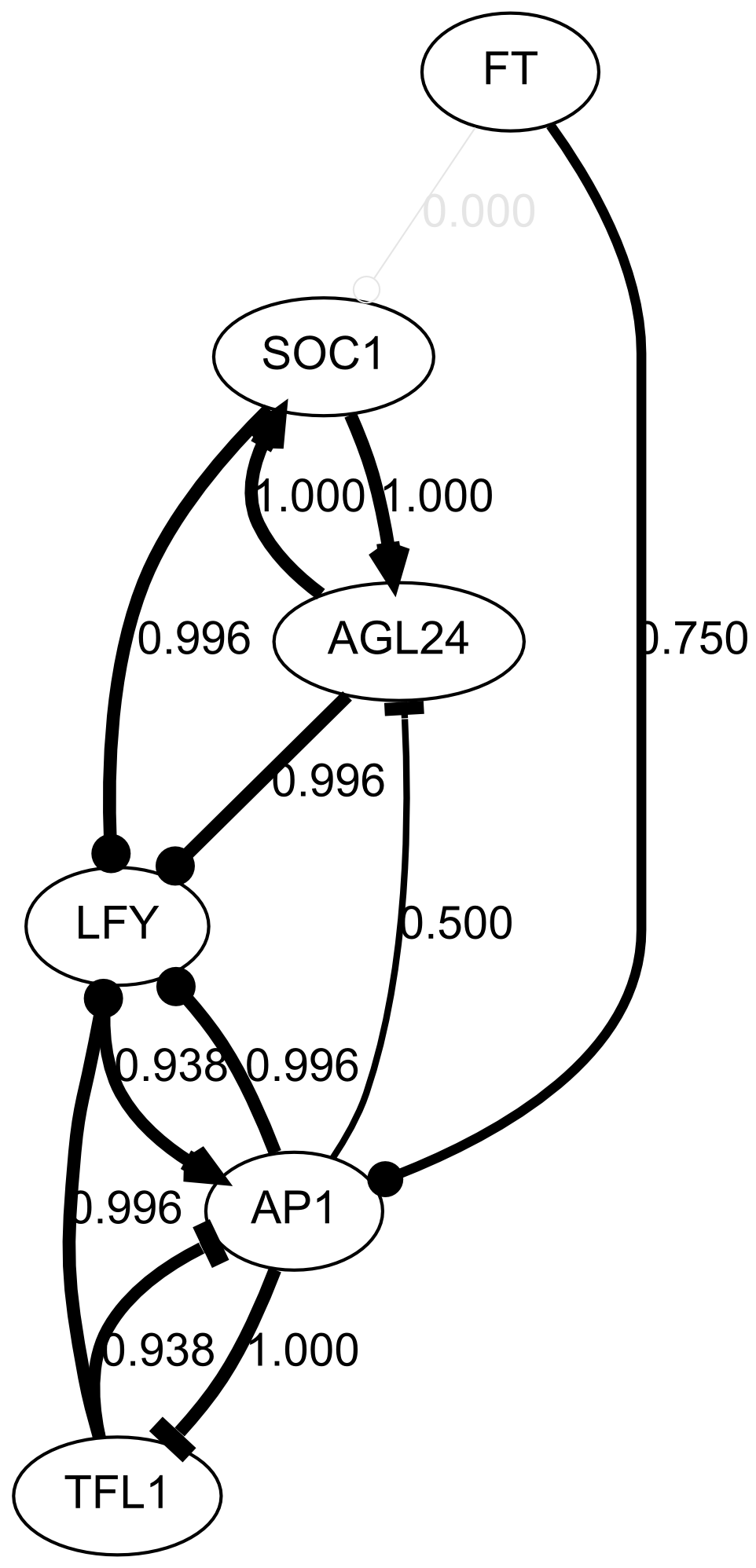

Supplement: S1 Fig — Nodes are genes. Edges represent regulatory interactions. Edge labels and edge thicknesses denote the occurrence frequencies of the associated interactions. V-, T- and O-shaped arrowheads indicate positive, negative and ambiguous interactions, respectively. (TIF) [file pcbi.1005744.s001.tif]

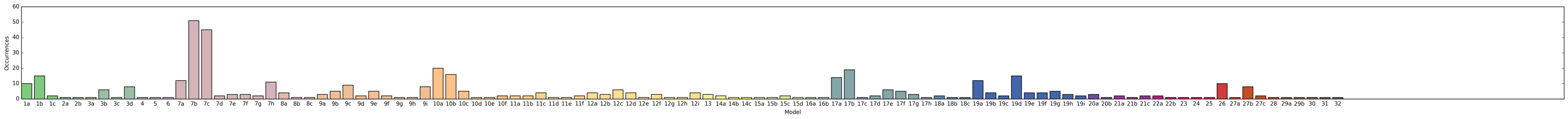

Supplement: S2 Fig — Models with the same number have the same fitness value. (PNG) [file pcbi.1005744.s002.png]
