# Supplementary material for: The logic of the floral transition: Reverse-engineering the switch controlling the identity of lateral organs
Source: PLoS Comput Biol. 2017 Sep 20;13(9):e1005744. doi: 10.1371/journal.pcbi.1005744 (PMC5624648; doi:10.1371/journal.pcbi.1005744)
Supplement: S1 Table — Dates are expressed as days after germination (dag), days after induction (dai) or as developmental stages when no other information was available (vegetative, transition or inflorescence). (PDF) [file pcbi.1005744.s003.pdf]

Genes and times	Reference
FD (6, 8, 10 dag)<br><br>*SOC1* (6, 10 dag)	[1]
TFL1 (7, 14, 17 dag; inflorescence)<br><br>AP1 (inflorescence)<br><br>LFY (inflorescence)	[2]
FD (0, 4, 5, 6 dai)<br><br>AP1 (0, 4, 5, 6 dai)	[3]
TFL1 (12 dag)<br><br>TFL1 protein (12, 16 dag)	[4]
AP1 (inflorescence)<br><br>SOC (inflorescence)	[5]
SOC1 (0, 1, 3, 5 dai)<br><br>AP1 (0, 3, 5 dai)	[6]
AGL24 (inflorescence)	[7]
LFY (inflorescence)	[8]

1. Searle I, He Y, Turck F, Vincent C, Fornara F, Kröber S, et al. The transcription factor FLC confers a flowering response to vernalization by repressing meristem competence and systemic signaling in Arabidopsis. Genes Dev. 2006 Apr 1;20(7):898–912.

2. Liu C, Teo ZWN, Bi Y, Song S, Xi W, Yang X, et al. A Conserved Genetic Pathway Determines Inflorescence Architecture in Arabidopsis and Rice. Dev Cell. 2013 Mar 25;24(6):612–22.

3. Wigge PA, Kim MC, Jaeger KE, Busch W, Schmid M, Lohmann JU, et al. Integration of Spatial and Temporal Information During Floral Induction in Arabidopsis. Science. 2005 Aug 12;309(5737):1056–9.

4. Conti L, Bradley D. TERMINAL FLOWER1 Is a Mobile Signal Controlling Arabidopsis Architecture. Plant Cell. 2007 Mar;19(3):767–78.

5. Liu C, Zhou J, Bracha-Drori K, Yalovsky S, Ito T, Yu H. Specification of Arabidopsis floral meristem identity by repression of flowering time genes. Development. 2007 May 15;134(10):1901–10.

6. Wang J-W, Czech B, Weigel D. miR156-Regulated SPL Transcription Factors Define an Endogenous Flowering Pathway in Arabidopsis thaliana. Cell. 2009 Aug 21;138(4):738–49.

7. Michaels SD, Ditta G, Gustafson-Brown C, Pelaz S, Yanofsky M, Amasino RM. AGL24 acts as a promoter of flowering in Arabidopsis and is positively regulated by vernalization. Plant J. 2003 Mar 1;33(5):867–74.

8. Blazquez MA, Soowal LN, Lee I, Weigel D. LEAFY expression and flower initiation in Arabidopsis. Development. 1997 Oct 1;124(19):3835–44.
