# Supplementary material for: The logic of the floral transition: Reverse-engineering the switch controlling the identity of lateral organs
Source: PLoS Comput Biol. 2017 Sep 20;13(9):e1005744. doi: 10.1371/journal.pcbi.1005744 (PMC5624648; doi:10.1371/journal.pcbi.1005744)
Supplement: S2 Table — (PDF) [file pcbi.1005744.s004.pdf]

Rank	Equations	Graph
1a	TFL1 protein' = or(TFL1, TFL1 protein)<br>Auxin pathway' = not(or(TFL1, apex))<br>FD' = not(AP1)<br>SOC1' = and(or(FT, AGL24), FD)<br>AGL24' = SOC1<br>LFY' = and(or(not(TFL1 protein), Auxin pathway), or(and(AGL24, Auxin), AP1))<br>AP1' = and(LFY, not(TFL1 protein))<br>TFL1' = and(inner, not(AP1))	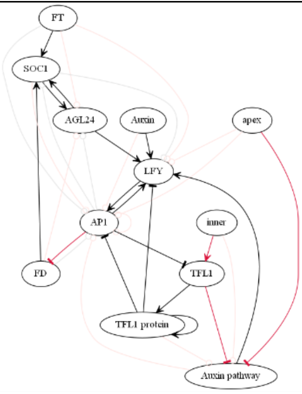
1b	TFL1 protein' = or(TFL1 protein, TFL1)<br>Auxin pathway' = not(or(apex, TFL1))<br>FD' = not(AP1)<br>SOC1' = and(or(AGL24, FT), FD)<br>AGL24' = SOC1<br>LFY' = and(or(AP1, and(SOC1, Auxin)), or(not(TFL1 protein), Auxin pathway))<br>AP1' = and(LFY, not(TFL1 protein))<br>TFL1' = and(inner, not(AP1))	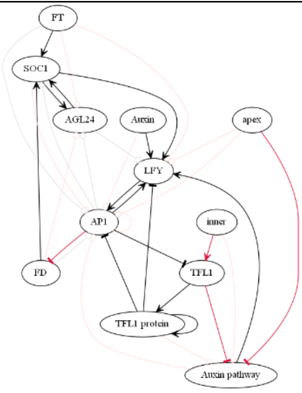

1c	TFL1 protein' = or(TFL1 protein, TFL1)<br>Auxin pathway' = not(or(TFL1, apex))<br>FD' = not(AP1)<br>SOC1' = and(or(AGL24, FT), FD)<br>AGL24' = SOC1<br>LFY' = or(AP1, and(and(Auxin, AGL24), or(not(TFL1 protein), Auxin pathway)))<br>AP1' = and(not(TFL1 protein), LFY)<br>TFL1' = and(inner, not(AP1))	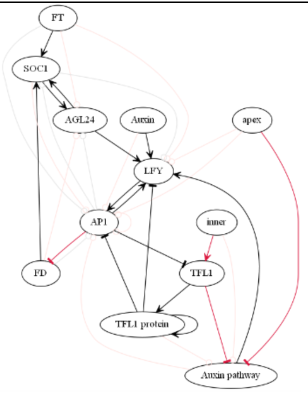
2a	TFL1 protein' = or(TFL1, TFL1 protein)<br>Auxin pathway' = not(or(apex, TFL1))<br>FD' = not(AP1)<br>SOC1' = or(and(FD, FT), and(FD, AGL24))<br>AGL24' = SOC1<br>LFY' = or(and(or(not(AGL24), Auxin pathway), and(SOC1, Auxin)), AP1)<br>AP1' = and(not(TFL1 protein), LFY)<br>TFL1' = and(inner, not(AP1))	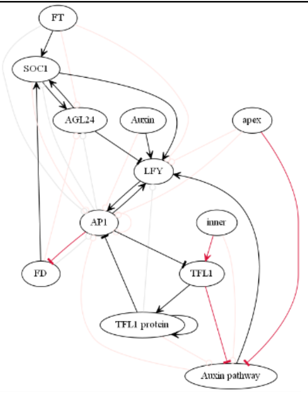

2b	TFL1 protein' = or(TFL1, TFL1 protein)<br><br>Auxin pathway' = not(or(TFL1, apex))<br><br>FD' = not(AP1)<br><br>SOC1' = or(and(FD, AGL24), and(FT, FD))<br><br>AGL24' = SOC1<br><br>LFY' = or(and(SOC1, and(Auxin, or(not(TFL1 protein), Auxin pathway))), AP1)<br><br>AP1' = and(not(TFL1 protein), LFY)<br><br>TFL1' = and(inner, not(AP1))	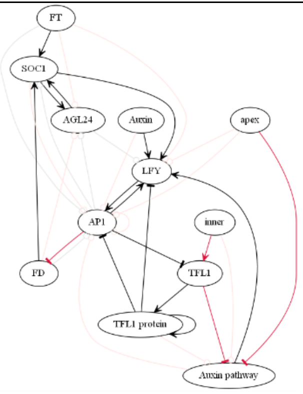
3a	TFL1 protein' = or(TFL1 protein, TFL1)<br><br>Auxin pathway' = not(or(TFL1, apex))<br><br>FD' = not(AP1)<br><br>SOC1' = or(AGL24, and(FT, FD))<br><br>AGL24' = and(not(AP1), SOC1)<br><br>LFY' = or(AP1, and(or(Auxin pathway, not(TFL1 protein)), and(SOC1, Auxin)))<br><br>AP1' = and(LFY, not(TFL1 protein))<br><br>TFL1' = and(inner, not(AP1))	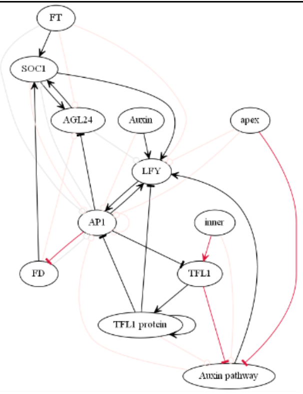

3b	TFL1 protein' = or(TFL1, TFL1 protein)<br><br>Auxin pathway' = not(or(TFL1, apex))<br><br>FD' = not(AP1)<br><br>SOC1' = or(and(FT, FD), AGL24)<br><br>AGL24' = and(not(AP1), SOC1)<br><br>LFY' = and(or(AP1, and(Auxin, AGL24)), or(not(TFL1 protein), Auxin pathway))<br><br>AP1' = and(not(TFL1 protein), LFY)<br><br>TFL1' = and(inner, not(AP1))	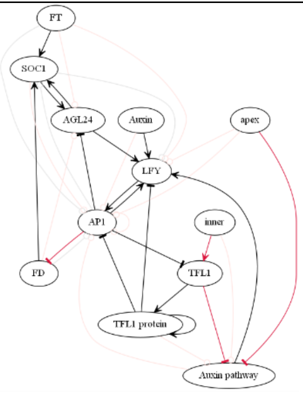
3c	TFL1 protein' = or(TFL1 protein, TFL1)<br><br>Auxin pathway' = not(or(TFL1, apex))<br><br>FD' = not(AP1)<br><br>SOC1' = or(AGL24, and(FD, FT))<br><br>AGL24' = and(SOC1, not(AP1))<br><br>LFY' = or(AP1, and(and(AGL24, or(Auxin pathway, not(TFL1 protein))), Auxin))<br><br>AP1' = and(not(TFL1 protein), LFY)<br><br>TFL1' = and(inner, not(AP1))	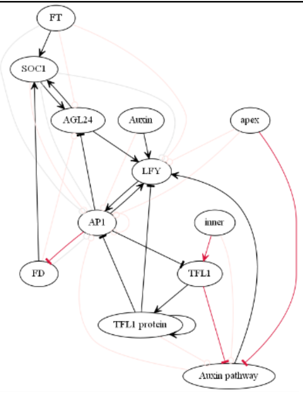

<table>
<tr><td>3d</td><td>TFL1 protein' = or(TFL1 protein, TFL1)

Auxin pathway' = not(or(TFL1, apex))

FD' = not(AP1)

SOC1' = or(and(FT, FD), AGL24)

AGL24' = and(SOC1, not(AP1))

LFY' = and(or(and(Auxin, SOC1), AP1), or(Auxin pathway, not(TFL1 protein)))

AP1' = and(LFY, not(TFL1 protein))

TFL1' = and(inner, not(AP1))</td><td>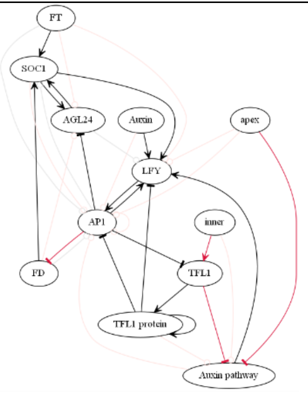
</td></tr>
<tr><td>4</td><td>TFL1 protein' = or(TFL1 protein, TFL1)

Auxin pathway' = not(or(apex, TFL1))

FD' = not(AP1)

SOC1' = and(FD, or(FT, AGL24))

AGL24' = SOC1

LFY' = and(not(not(or(and(Auxin, SOC1), AP1))), or(and(AGL24, Auxin pathway), not(TFL1 protein)))

AP1' = and(not(TFL1 protein), LFY)

TFL1' = and(inner, not(AP1))</td><td>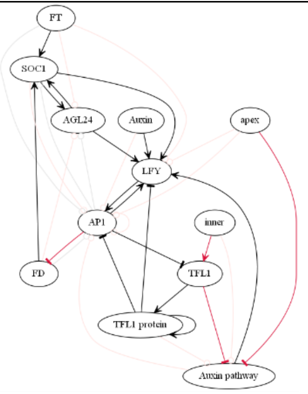
</td></tr>
</table>

<table>
<tr>
<td>5</td>
<td>TFL1 protein' = or(TFL1 protein, TFL1)

Auxin pathway' = not(or(TFL1, apex))

FD' = not(AP1)

SOC1' = or(and(FD, FT), AGL24)

AGL24' = and(not(AP1), SOC1)

LFY' = or(and(Auxin, or(and(Auxin pathway, AGL24), and(SOC1, not(TFL1 protein)))), AP1)

AP1' = and(not(TFL1 protein), LFY)

TFL1' = and(inner, not(AP1))</td>
<td>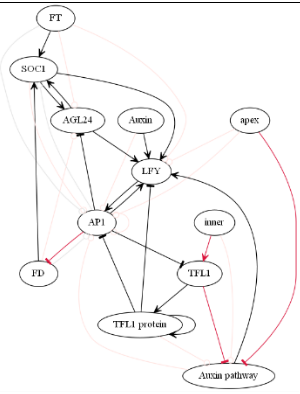
</td>
</tr>
<tr>
<td>6</td>
<td>TFL1 protein' = or(TFL1, TFL1 protein)

Auxin pathway' = not(or(TFL1, apex))

FD' = not(AP1)

SOC1' = or(and(FT, FD), AGL24)

AGL24' = and(SOC1, not(AP1))

LFY' = and(or(AP1, and(Auxin, SOC1)), or(Auxin pathway, not(AGL24)))

AP1' = not(and(FD, or(not(LFY), TFL1 protein)))

TFL1' = and(inner, not(AP1))</td>
<td>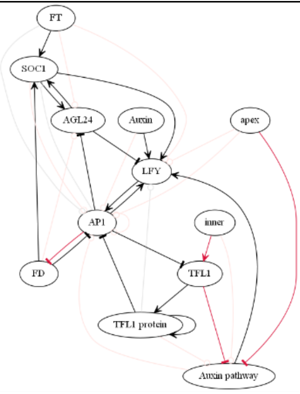
</td>
</tr>
</table>
