# Supplementary material for: The logic of the floral transition: Reverse-engineering the switch controlling the identity of lateral organs
Source: PLoS Comput Biol. 2017 Sep 20;13(9):e1005744. doi: 10.1371/journal.pcbi.1005744 (PMC5624648; doi:10.1371/journal.pcbi.1005744)
Supplement: S1 Text — (PDF) [file pcbi.1005744.s005.pdf]

Boolean modelling

Boolean networks

Boolean network models represent genes as binary variables (either on or off) that influence each other dynamically, following a specified set of logical rules that can be written using combinations of the AND, OR and NOT operators [35]. The state of the network (i.e.: of each gene) at a given time point depends on the state of the network at the previous time point. The function that yields the next state of the model when given any state as an input is called the successor function, and that next state is called the successor of the state given as input.

As the number of states of a network is finite, a chain of successors starting at any state will sooner or later include at least one state more than once and initiate a periodic pattern. That periodic pattern constitutes an attractor. If the chain ends with the repetition of a single successor, this attractor is a steady state. If the chain ends with the repetition of multiple states, this attractor is a cycle.

Another consequence of the number of states being finite is that it is possible to establish an exhaustive list of states and their successors, for any model (or part of a model). This list is usually presented as a truth table, which is a table divided into a left-hand side and a right hand side. The left hand side lists all the possible states of the regulators of the genes of interest, while the right-hand side contains the matching states of the genes of interests at the following time step.

Updating scheme

Synchronous updating (i.e.: multiple variables can change their values per time step) was chosen over asynchronous updating (i.e.: only one variable can change its value per time step). Asynchronous updating is generally considered more realistic, as, in reality, time is continuous, so multiple genes are unlikely to change their states at once. However, asynchronous updating

has a drawback: when multiple genes might change their states at once, the Boolean states typically have multiple potential successors, leading to non-deterministic outcomes. Furthermore, steady states are independent of the updating scheme.
