# Supplementary material for: The logic of the floral transition: Reverse-engineering the switch controlling the identity of lateral organs
Source: PLoS Comput Biol. 2017 Sep 20;13(9):e1005744. doi: 10.1371/journal.pcbi.1005744 (PMC5624648; doi:10.1371/journal.pcbi.1005744)
Supplement: S2 Text — (PDF) [file pcbi.1005744.s006.pdf]

Exhaustive search

Since each model is characterized by a truth table, exhaustive search works by enumerating all possible truth tables. Here, to make the problem more tractable, we restrict the set of possible truth tables to those compatible with the topology of a reference network established from biological data.

As a starting point, truth tables with empty right-hand sides are generated for each internal node (i.e. node with at least one inbound edge) of the reference network, based on its topology. This means that, if C is regulated by A and B in the reference network, the truth table of C will have two columns corresponding to A and B on its left side, and one corresponding to C on its right side. This ensures that an interaction will be in the model only if it is in the reference network, which is our criterion for the consistency of a model with the reference network topology. Interactions that are in the predefined network are not required to be in a model for this model to be considered consistent: this is to account for the fact that interactions might be "biologically real" but of low magnitude, thereby not justifying their inclusion in a Boolean model. Truth tables will then be enumerated by completing the left-hand sides in all possible ways.

However, some of these combinations can be ruled out without any calculation, because they are not compatible with the required steady states. This leverages the fact that, for a steady state S, successor(S) = S. Therefore, if the left side of a row of a truth table matches S, then its right side should also match S. This means some rows of the right-hand side of the truth tables can be prefilled before the actual enumeration.

At this stage, an incompatibility between two steady states can occur (i.e. the left side of a row matches two steady states A and B, but A and B contain conflicting information regarding how the right side of the row should be prefilled), in which case the search problem has no solution.

Assuming steady states A and B are correct, it means that the topology of the reference network is incorrect, possibly due to missing interactions. Adding interactions such that A and B no longer match the same row of the left side of the truth table might resolve the conflict.

If all steady states are compatible, we proceed to iterate over all possible values for the empty cells of the truth tables, and record the models that have all the required transitions as valid, if any have been defined. If not, then all models are considered valid.

The actual enumeration is performed in a naïve way: all possible sequences of values (0/1, or ON/OFF) are considered. Consistence with the reference network topology is already guaranteed by the structure of the truth tables. The signs of interactions (activation/repression) are ignored when assessing consistency. This is to reflect that the techniques used to identify direct interactions are mostly ChIP-related, and cannot determine the signs of interactions. Complementary approaches such as genetic modification can somewhat address that limitation, however they only show the effect of a transcription factor in a very specific context, determined by the combination of other proteins also present. It is possible that the same transcription factor would have a different effect in presence of another combination of proteins.

The assessment of the presence or absence of potential interactions – and their signs if applicable – is performed after the search, in a separate analysis step (S6 Text).
