# Supplementary material for: The logic of the floral transition: Reverse-engineering the switch controlling the identity of lateral organs
Source: PLoS Comput Biol. 2017 Sep 20;13(9):e1005744. doi: 10.1371/journal.pcbi.1005744 (PMC5624648; doi:10.1371/journal.pcbi.1005744)
Supplement: S3 Text — (PDF) [file pcbi.1005744.s007.pdf]

Verifying if a model can explain a transition

Transitions are implemented as tuples (I, P, F, C, M) where:

- I is an initial steady state
- P is a perturbation to be applied to I. The resulting state is P(I).
- F is the steady state that P(I) is supposed to lead to, if it is left to evolve spontaneously. For a given model m, if $attractor_m(P(I)) = F$, then the model can explain this transition. If $attractor_m(P(I))$ is a cycle, model m is rejected, even if $attractor_m(P(I))$ contains F.
- C is a certainty mask used to modulate the comparison between $attractor_m(P(I))$ and F. It is a Boolean vector of the same size as I and F. 1 values in C indicate the associated variables in $attractor_m(P(I))$ and F should be taken into account for the comparison, 0 values mean they should not. This means model m can explain the transition if all variables in ($attractor_m(P(I))$ XOR F) AND C are 0.
- M is a list of mutations. If it is not empty, the transition should apply to a "mutant variant" of model m (instead of applying to model m directly).
