# Supplementary material for: The logic of the floral transition: Reverse-engineering the switch controlling the identity of lateral organs
Source: PLoS Comput Biol. 2017 Sep 20;13(9):e1005744. doi: 10.1371/journal.pcbi.1005744 (PMC5624648; doi:10.1371/journal.pcbi.1005744)
Supplement: S4 Text — (PDF) [file pcbi.1005744.s008.pdf]

Modeling of mutants

Let f be the successor function of a WT model, and f_i the function giving its i-th component. Let f^j be the successor function of the same model, with a knock-out mutation of species j, and f_i^j the function yielding its i-th component. Let X be a state of the system.

$$\forall i \neq j, \qquad \forall X, \qquad f_i^j(X) = f_i(X)$$

$$f_j^j(X) = 0$$
