# Supplementary material for: The logic of the floral transition: Reverse-engineering the switch controlling the identity of lateral organs
Source: PLoS Comput Biol. 2017 Sep 20;13(9):e1005744. doi: 10.1371/journal.pcbi.1005744 (PMC5624648; doi:10.1371/journal.pcbi.1005744)
Supplement: S5 Text — (PDF) [file pcbi.1005744.s009.pdf]

Genetic programming

Genetic programming is a method to generate structured sequences like computer code or mathematic equations using a genetic algorithm. We applied this method to the generation of Boolean models using the DEAP module [1] in Python.

Structured sequences can be written as a tree. In the case of equations, a node is a function, and the children of that node are its arguments.

In genetic programming, the nodes of the trees are called primitives.

Primitives

In our case, the leaf primitives are the nodes of the GRN. They can be combined into Boolean expressions using AND, OR and NOT nodes. Finally, at the top level, Boolean expressions are aggregated into a list of Boolean expressions (one expression for each state variable of the model).

Primitive	Type	Arguments
List maker	List	6 bools
And	Bool	2 bools
Or	Bool	2 bools
Not	Bool	1 bool
Nodes of the GRN	Bool	None

Offspring generation

At each iteration of the algorithm, as many offspring individuals as there are individuals in the original population, using mutation, mating or reproduction.

1.1.1.1 Mutation

A branch of the tree is replaced with a random branch. This occurs with probability 0.4.

1.1.1.2 Mating

Exchange of branches of the tree related to the same genes. This occurs with probability 0.4.

1.1.1.3 Reproduction

An individual is copied as-is. This occurs with probability 0.2.

Constraint on the trees

The maximal depth of the tree is capped to 11 in order to avoid bloat. Lower values reduce the size of the search space, but if they are too low, they can prevent solutions from being found.

Selection

n individuals are selected out of the n original individuals and their n offspring individuals using n 2-invidual tournaments. The whole population is split randomly into n pairs, and the better individual of each pair is selected to be part of the next generation.

References

1. Fortin F-A, De Rainville F-M, Gardner M-AG, Parizeau M, Gagné C. DEAP: Evolutionary Algorithms Made Easy. J Mach Learn Res. 2012 Jul;13(1):2171–2175.
