# Supplementary material for: The logic of the floral transition: Reverse-engineering the switch controlling the identity of lateral organs
Source: PLoS Comput Biol. 2017 Sep 20;13(9):e1005744. doi: 10.1371/journal.pcbi.1005744 (PMC5624648; doi:10.1371/journal.pcbi.1005744)
Supplement: S6 Text — (PDF) [file pcbi.1005744.s010.pdf]

Model analysis

Adjacency matrix

For each state of the model, the effect of switching on inactive genes one at a time was recorded in an adjacency matrix whose values (-1, 0, 1 or 2) indicate the type of each regulatory interaction.

Let n be the number of species in the model. Let A be a $n \times n$ matrix. Let $X = (X_1, \dots, X_i, \dots, X_n)$ be a model state. Let $X_i^* = (X_1, \dots, 1, \dots, X_n)$ be a state derived from X by setting the value of the i-th node to 1. Let f be the successor function of the model. Let $Y = f(X) = (Y_1, \dots, Y_j, \dots, Y_n)$ and $Y_i^* = f(X_i^*) = ({Y_i^*}_1, \dots, {Y_i^*}_j, \dots, {Y_i^*}_n)$.

For each (i, j) pair:

- If, for all X, $Y_j = {Y_i^*}_j$, then $A_{ij} = 0$
- Else, if, for all X, $Y_j \le {Y_i^*}_j$, then $A_{ij} = 1$
- Else, if, for all X, $Y_j \ge {Y_i^*}_j$, then $A_{ij} = -1$
- Else, $A_{ij} = 2$

Aggregation of multiple models

The following method was used to aggregate the adjacency matrices of a set of models K into a single matrix.

Let A be the aggregated adjacency matrix and A^k the adjacency matrix of model k.

- If $\forall k \in K,\ A_{ij}^k = 0$, then $A_{ij} = 0$.
- If $\forall k \in K,\ A_{ij}^k \in \{0, 1\}$, then $A_{ij} = 1$.
- If $\forall k \in K,\ A_{ij}^k \in \{-1, 0\}$, then $A_{ij} = -1$.
- If $\exists k \in K,\ A_{ij}^k = -1$ and $\exists k' \in K,\ A_{ij}^{k'} = 1$, then $A_{ij} = 2$.

- If $\exists k \in K,\ A_{ij}^{k} = 2$, then $A_{ij} = 2$.

Graph representation

Let A be the adjacency matrix of a model and Gits graph.

- If $A_{ij} \neq 0$, G includes the regulatory edge $i \rightarrow j$.
- If $A_{ij} = 1$, i is an activator of j.
- If $A_{ij} = -1$, i is a repressor of j.
- If $A_{ij} = 2$, the effect of i on j is ambiguous.

Principal components analysis (PCA)

PCA was carried out on sets of models to assess their diversity.

First, a matrix of Boolean values was built, where:

- Each row is a row vector of Boolean variables indicating which interactions are present in a model;
- Each column corresponds to a directed interaction edge in the GRN. Only interactions that vary in the set of models are retained (the variance of the column vector is greater than 0).

PCA was then performed using the Scikit-learn module [1] for Python.

References

1. Pedregosa F, Varoquaux G, Gramfort A, Michel V, Thirion B, Grisel O, et al. Scikit-learn: Machine Learning in Python. J Mach Learn Res. 2011 Oct;12:2825−2830.
